# Mechanistic Insight into the Degradation of Nitrosamines via Aqueous-Phase UV Photolysis or a UV-Based Advanced Oxidation Process: Quantum Mechanical Calculations

**DOI:** 10.3390/molecules23030539

**Published:** 2018-02-28

**Authors:** Daisuke Minakata, Erica Coscarelli

**Affiliations:** Department of Civil and Environmental Engineering, Michigan Technological University, 1400 Townsend Drive, Houghton, MI 49931, USA; eacoscar@mtu.edu

**Keywords:** nitrosamines, NDMA, hydroxyl radicals, UV photolysis, advanced oxidation processes, quantum mechanical calculation

## Abstract

Nitrosamines are a group of carcinogenic chemicals that are present in aquatic environments that result from byproducts of industrial processes and disinfection products. As indirect and direct potable reuse increase, the presence of trace nitrosamines presents challenges to water infrastructures that incorporate effluent from wastewater treatment. Ultraviolet (UV) photolysis or UV-based advanced oxidation processes that produce highly reactive hydroxyl radicals are promising technologies to remove nitrosamines from water. However, complex reaction mechanisms involving radicals limit our understandings of the elementary reaction pathways embedded in the overall reactions identified experimentally. In this study, we perform quantum mechanical calculations to identify the hydroxyl radical-induced initial elementary reactions with *N*-nitrosodimethylamine (NDMA), *N*-nitrosomethylethylamine, and *N*-nitrosomethylbutylamine. We also investigate the UV-induced NDMA degradation mechanisms. Our calculations reveal that the alkyl side chains of nitrosamine affect the reaction mechanism of hydroxyl radicals with each nitrosamine investigated in this study. Nitrosamines with one- or two-carbon alkyl chains caused the delocalization of the electron density, leading to slower subsequent degradation. Additionally, three major initial elementary reactions and the subsequent radical-involved reaction pathways are identified in the UV-induced NDMA degradation process. This study provides mechanistic insight into the elementary reaction pathways, and a future study will combine these results with the kinetic information to predict the time-dependent concentration profiles of nitrosamines and their transformation products.

## 1. Introduction 

Nitrosamines, which contain N–NO functional groups, are a group of chemicals that pose mutagenicity, teratogenicity, and carcinogenicity [1]. Nitrosamines are the byproducts of various manufacturing, agricultural, and natural processes and have been found in natural aquatic environments and in the effluent of wastewater treatment processes [2]. As a type of nitrosamine, *N*-nitrosodimethyl amine (NDMA, (CH_3_)_2_N–NO) is a low-molecular-weight, neutral, organic contaminant that has also been found to be present in aquatic environments. The California Department of Health Services has set notification levels of 10 ng/L for NDMA and other nitrosamines in drinking water [3]. 

Ultraviolet (UV) photolysis and UV-based advanced oxidation processes (AOPs) that produce highly reactive hydroxyl radicals (HO^•^) are attractive and promising water treatment technologies, which can inactivate pathogens and destroy a wide variety of organic chemical contaminants [4,5]. UV photolysis and UV-AOPs have been employed in wastewater reclamation processes for indirect or direct potable reuse of treated wastewater to increase water security and address water scarcity issues in many arid regions [6]. Wastewater reclamation processes use multiple barriers to physically remove pathogens and chemical contaminants via microfiltration/ultrafiltration, followed by nanofiltration (NF)/reverse osmosis (RO). After the NF/RO process, UV photolysis or UV-AOPs inactivate pathogens and destroy chemicals present in the NF/RO permeate stream. Over 50% of NDMA has been found to be present in the NF/RO permeate, and the use of UV photolysis or UV-AOPs are necessary to remove NDMA and other nitrosamines [7].

UV photolysis using a low-pressure UV lamp that emits photons at a wavelength of 254 nm is very effective at destroying NDMA due to the high molar absorptivity (1650 M^−1^cm^−1^ at 253.7 nm) and highly reactive HO^•^ produced in AOPs rapidly react with many nitrosamines to effectively destroy the initial contaminants (the second order reaction rate constants of HO^•^; *k* = 10^8^–10^9^ M^−1^s^−1^) [8,9]. However, complex chemical reactions involving radicals produce a number of transformation byproducts, and hence, detailed reactivity and reaction pathways for NDMA and other nitrosamines have not been elucidated yet. For example, Mezyk’s group studied the kinetics of HO^•^ with various structurally different nitrosamines, and found that NDMA, *N*-nitrosomethylethylamine (NMEA) and *N*-nitrosodiethylamine (NDEA) showed different reactivity and degradation efficiency from other nitrosamines that have longer alkyl chains adjacent to the N–NO functional group. They proposed that radical delocalization caused the differences in the degradation efficiency, but the detailed reaction pathway has not been identified yet [8]. Stefan and Bolton (2002) investigated reaction pathways for NDMA degradation based on laboratory-scale batch photolysis experiments and explained the initial photolysis mechanisms based on the reaction pathways previously identified by studies in the 1960s and 1970s [10,11,12,13,14]. UV-induced NDMA degradation pathways were studied at both pH 3 and pH 7 to identify the transformation products, such as methylamine, dimethylamine, formaldehyde, formic acid, nitrite ion and nitrate ion [15,16]. Their careful experiments and measurement of transformation products proposed several key reaction pathways that were induced by UV photolysis at a wavelength of 253.7 nm at different pH values [15,16]. However, some of the pathways involved in the formation of transformation products are still unknown. UV-induced NDMA degradation was also studied and identified previously unknown reactive species in the NDMA degradation pathways [17,18,19]. The HO^•^-induced NDMA degradation mechanisms were studied in an ozone-based AOP, and general reaction mechanisms were proposed [20,21]. The major transformation mechanisms were proposed based on experimental studies of the products, but the elementary reaction pathways are not known due to difficulties in identifying the embedded reactions that were involved in the overall reaction.

Quantum mechanical (QM) calculations using *ab initio* methods or density functional theory (DFT) are attractive approaches to identify elementary reaction pathways and the kinetics of complex fast radical reactions [22]. QM calculations have been used to support experimentally identified reaction pathways by calculating the reaction energy using statistical thermodynamics. Aqueous-phase enthalpy and free energies of activation and reaction were calculated to determine the dominant degradation pathway of dimethyl phthalate [23]. Elementary reactions involved in the HO^•^-induced mineralization of flutriafol were identified [24]. DFT calculations were used to determine the NDMA formation mechanism from *N*,*N*-dimethylsulfamide via ozonation in water [25]. A high-level multi-point energy method was used to calculate the aqueous-phase free energies of activation for HO^•^-induced reactions of a wide variety of organic compounds, including aliphatic compounds, alkenes, and aromatic compounds [26,27,28]. These studies highlight the usefulness of QM-based calculations to provide insight into reaction mechanisms that cannot be obtained by experiments. In addition, the findings from QM-based calculations also provide potential transformation products that can be identified in future experiments. 

In this study, we use QM-based calculations to identify the HO^•^-induced initial elementary reactions with NDMA and other nitrosamines as well as the UV-induced NDMA degradation pathways at 254 nm of wavelength. We investigate NDMA, NMEA, and *N*-nitrosomethylbutylamine (NMBA), which have different alkyl side chains that are adjacent to the nitroso functional group (–N–NO), to elucidate the effect of the alkyl side chain on the overall reactivity with HO^•^. We also investigate UV-induced NDMA degradation using time-dependent (TD)-DFT to understand the molecular orbitals responsible for electron excitation and the nitrogen-containing radical reactions during the photolysis of NDMA.

## 2. Results and Discussion

### 2.1. HO^•^-Induced Degradation

#### 2.1.1. *N*-Nitrosodimethylamine (NDMA) Degradation Pathways Induced by HO^•^

**Figure 1:**
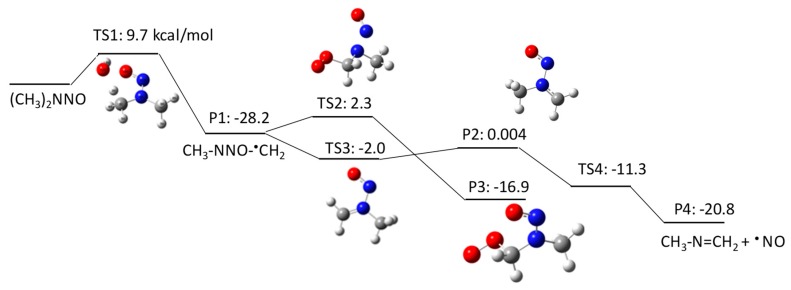
Free energy profile for pathway 1–1 of the HO^•^-induced reaction pathways for *N*-nitrosodimethylamine (NDMA) via H abstraction. TS denotes the transition state, and P denotes the product. The numbers (kcal/mol) are the free energy of activation for the TS and free energy of reaction for the P relative to the corresponding reactant.

**Figure 2:**
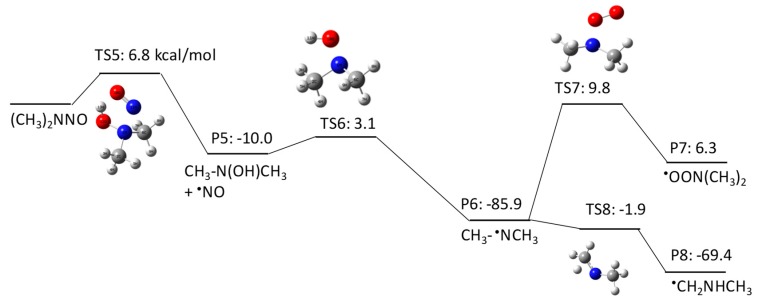
Free energy profile for pathway 1–2 of the HO^•^-induced reaction pathways for *N*-nitrosodimethylamine (NDMA) via HO^•^ addition to amine nitrogen. The numbers (kcal/mol) are the free energy of activation for the TS and free energy of reaction for the P relative to the corresponding reactant.

**Figure 3:**
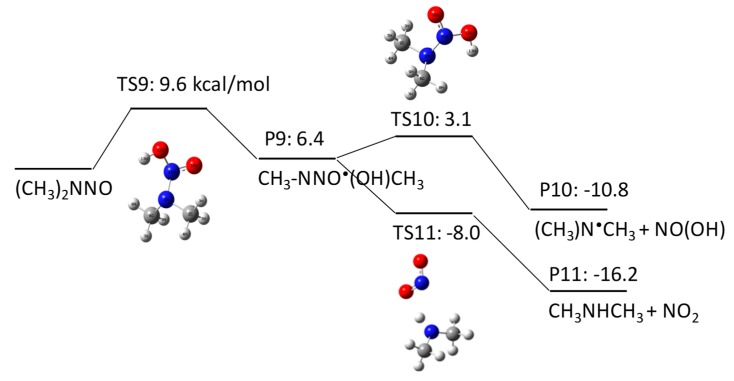
Free energy profile for pathway 1–3 of the HO^•^-induced reaction pathways for *N*-nitrosodimethylamine (NDMA) via HO^•^ addition to the nitrosyl nitrogen. The numbers (kcal/mol) are the free energy of activation for the TS and free energy of reaction for the P relative to the corresponding reactant.

NDMA has three potential initial degradation mechanisms: (1) H atom abstraction from a C–H bond of the methyl group (pathway 1–1 in Figure 1), (2) HO^•^ addition to amine nitrogen (pathway 1–2 in Figure 2), and (3) HO^•^ addition to nitrosyl nitrogen (pathway 1–3 in Figure 3). Our QM calculations obtained $\Delta G_{\mathrm{aq},\mathrm{calc}}^{\mathrm{act}}$ values of 9.7 kcal/mol, 6.8 kcal/mol, and 9.6 kcal/mol for the respective pathways. H abstraction from a C–H bond forms a C-centered radical that reacts with the triplet state of molecular oxygen dissolved in water. Our previous studies indicate that the addition of molecular oxygen to a C-centered radical is a barrierless reaction with a $\Delta G_{\mathrm{aq},\mathrm{calc}}^{\mathrm{act}}$ of −20–30 kcal/mol, which enabled us to consistently predict the experimentally measured reaction rate constants [28]. The $\Delta G_{\mathrm{aq},\mathrm{calc}}^{\mathrm{act}}$ value obtained for the ^•^CH_2_NNOCH_3_ radical was 2.3 kcal/mol, which is significantly larger than those of typically observed reactions. This indicates that the N–NO functional group significantly affects molecular addition to the C-centered radical. The second-order reaction rate constant for the addition of molecular oxygen to a C-centered radical of NDMA was determined to be (5.3 ± 0.6) × 10^6^ M^−1^s^−1^ [9], which is three orders of magnitude smaller than the typically observed rate constants (~5 × 10^9^ M^−1^s^−1^) [29]. A more detailed discussion on the unique reactivity of molecular oxygen to C-centered radicals will be given in a later section. According to our calculations, the C-centered radical also undergoes electron transfer to produce CH_3_NNO=CH_2_ ($\Delta G_{\mathrm{aq},\mathrm{calc}}^{\mathrm{act}}$ of −2.0 kcal/mol), followed by the loss of NO^•^ ($\Delta G_{\mathrm{aq},\mathrm{calc}}^{\mathrm{act}}$ of −11.3 kcal/mol) to produce *N*-methylidenemethylamine (CH_2_=NHCH_3_). This latter pathway involves several barrierless reactions, and is dominant over the pathway involving the addition of molecular oxygen. The formation of *N*-methylidenemethylamine was also postulated in a previous report [18,19].

The second pathway is HO^•^ addition to the amine nitrogen, followed by the loss of an OH group. Although initial HO^•^ addition has a lower free energy of activation ($\Delta G_{\mathrm{aq},\mathrm{calc}}^{\mathrm{act}}$ of 6.8 kcal/mol) than the H abstraction identified in pathway 1–1, the subsequent reaction has a larger activation barrier ($\Delta G_{\mathrm{aq},\mathrm{calc}}^{\mathrm{act}}$ of 3.1 kcal/mol) to produce a *N*-centered radical (i.e., CH_3_^•^NCH_3_). The *N*-centered radical undergoes either molecular oxygen addition or an H shift. The H shift has a significantly smaller $\Delta G_{\mathrm{aq},\mathrm{calc}}^{\mathrm{act}}$ of −1.9 kcal/mol than molecular oxygen addition to the *N*-centered radical ($\Delta G_{\mathrm{aq},\mathrm{calc}}^{\mathrm{act}}$ of 9.8 kcal/mol). Thus, C-centered radical formation resulting from an H shift is the dominant pathway via TS8. The significantly large $\Delta G_{\mathrm{aq},\mathrm{calc}}^{\mathrm{act}}$ for the addition of molecular oxygen to a *N*-centered radical via TS7 can be verified by the experimentally obtained reaction rate constant for hydrazyl (*k* = 3.9 × 10^8^ M^−1^s^−1^) [30].

Pathway 1–3 involves initial HO^•^ addition to the nitrosyl nitrogen with a $\Delta G_{\mathrm{aq},\mathrm{calc}}^{\mathrm{act}}$ of 9.6 kcal/mol. Although this reaction has an almost identical $\Delta G_{\mathrm{aq},\mathrm{calc}}^{\mathrm{act}}$ to that of pathway 1–1, the initial HO^•^ addition reaction that produces an alkoxyl radical (i.e., CH_3_NNO^•^(OH)CH_3_) is not thermodynamically favored ($\Delta G_{\mathrm{aq},\mathrm{calc}}^{\mathrm{react}}$ of 6.4 kcal/mol). This alkoxyl radical undergoes two pathways to produce (1) a *N*-centered radical with a $\Delta G_{\mathrm{aq},\mathrm{calc}}^{\mathrm{act}}$ of 3.1 kcal/mol and (2) methyl diamine (CH_3_NHCH_3_) with a $\Delta G_{\mathrm{aq},\mathrm{calc}}^{\mathrm{act}}$ of −8.0 kcal/mol. 

The above investigation confirms that H abstraction from a C–H bond of the methyl functional group of NDMA is the dominant initial reaction pathway as induced by HO^•^, which is consistent with the experimental investigation using the electron paramagnetic resonance (ESR) technique [9]. The experimentally determined second-order rate constant was (4.3 ± 0.12) × 10^8^ M^−1^s^−1^, and this relatively slow H abstraction from a C–H bond by HO^•^ results from the electron-withdrawing effect of the neighboring N–NO functional group and the abnormally stable C-centered radical [9]. In the following sub-sections, the reactivity of NDMA will be compared to two other nitrosamines that have longer alkyl side chains (i.e., -CH_2_CH_3_ and -(CH_2_)_2_CH_3_) to investigate the unique reactivity of NDMA.

#### 2.1.2. *N*-Nitrosomethylethylamine (NMEA) Degradation Pathways Induced by HO^•^

**Figure 4:**
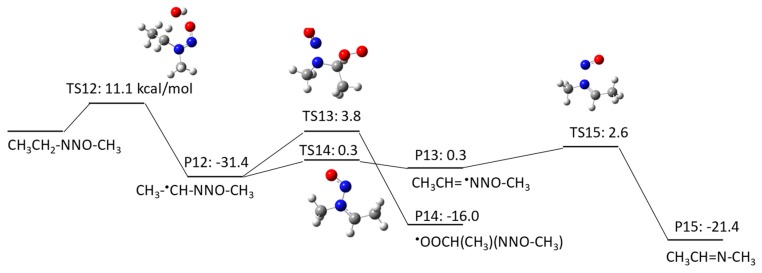
Free energy profile for pathway 2–1 of the HO^•^-induced reaction pathways for NMEA via H abstraction from a C–H bond of the –CH_2_– functional group adjacent to the N–NO functional group. The numbers (kcal/mol) are the free energy of activation for the TS and free energy of reaction for the P relative to the corresponding reactant.

**Figure 5:**
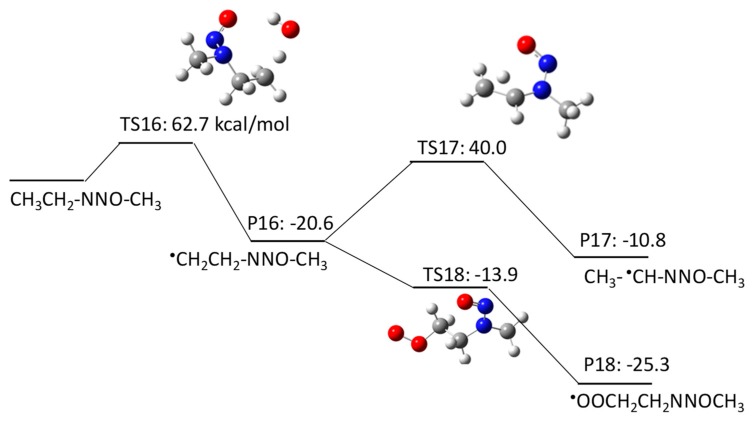
Free energy profile for pathway 2–2 of the HO^•^-induced reaction pathways for NMEA via H abstraction from a C–H bond of the terminal CH_3_ functional group in the ethyl chain. The numbers (kcal/mol) are the free energy of activation for the TS and free energy of reaction for the P relative to the corresponding reactant.

**Figure 6:**
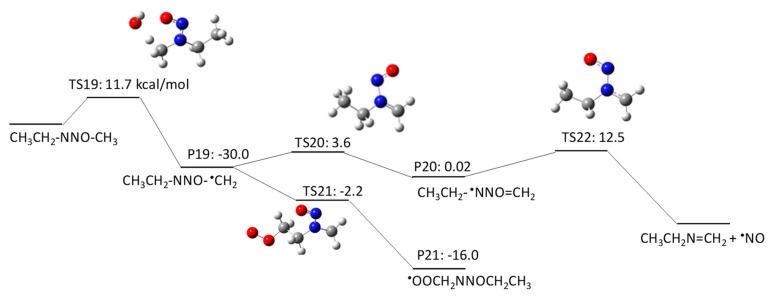
Free energy profile for pathway 2–3 of the HO^•^-induced reaction pathways for NMEA via H abstraction from a C–H bond of the terminal CH_3_ functional group adjacent to the N–NO functional group. The numbers (kcal/mol) are the free energy of activation for the TS and free energy of reaction for the P relative to the corresponding reactant.

NMEA has three potential H abstraction sites: (1) a C–H bond of the –CH_2_– functional group adjacent to the N–NO functional group by pathway 2–1; (2) a C–H bond of the terminal CH_3_ functional group in the ethyl chain by pathway 2–2; and (3) a C–H bond of the terminal CH_3_ functional group adjacent to the N–NO functional group by pathway 2–3. Figure 4, Figure 5 and Figure 6 show the free energy profiles per reaction coordinate for each pathway. Our calculations revealed similar $\Delta G_{\mathrm{aq},\mathrm{calc}}^{\mathrm{act}}$ values for H atom abstraction: 11.1 kcal/mol in pathway 2–1 and 11.7 kcal/mol in pathway 2–3), except 62.7 kcal/mol in pathway 2–2. It is still not clear why the pathway 2–2 had such a high barrier. All three pathways are thermodynamically favorable ($\Delta G_{\mathrm{aq},\mathrm{calc}}^{\mathrm{react}}$ < 0). Each pathway produces a C-centered radical, i.e., CH_3_^•^CHNNOCH_3_ in pathway 2–1, ^•^CH_2_CH_2_NNOCH_3_ in pathway 2–2, and CH_3_ CH_2_NNO^•^CH_2_ in pathway 2–3. The $\Delta G_{\mathrm{aq},\mathrm{calc}}^{\mathrm{act}}$ values for the addition of molecular oxygen to CH_3_^•^CHNNOCH_3_, ^•^CH_2_CH_2_NNOCH_3_, and CH_3_ CH_2_NNO^•^CH_2_ are 3.8 kcal/mol, −13.9 kcal/mol, and −2.2 kcal/mol, respectively. As observed in pathway 1, the $\Delta G_{\mathrm{aq},\mathrm{calc}}^{\mathrm{act}}$ values of these three C-centered radicals are still larger than the typical values (−20 to −25 kcal/mol). This indicates that the functional group directly neighboring the N–NO functional group affects the slow reaction of molecular oxygen addition to ^•^CH_2_CH_2_NNOCH_3_. Given that the other reaction pathways of the three C-centered radicals have either a larger $\Delta G_{\mathrm{aq},\mathrm{calc}}^{\mathrm{act}}$ than that for molecular oxygen addition or include thermodynamically unfavorable reactions ($\Delta G_{\mathrm{aq},\mathrm{calc}}^{\mathrm{react}}$ > 0), the formation of peroxyl radicals resulting from the addition of molecular oxygen is the dominant reaction pathway in the subsequent NMEA degradation mechanism.

#### 2.1.3. *N*-Nitrosomethylbutylamine (NMBA) Degradation Pathways Induced by HO^•^

**Figure 7:**
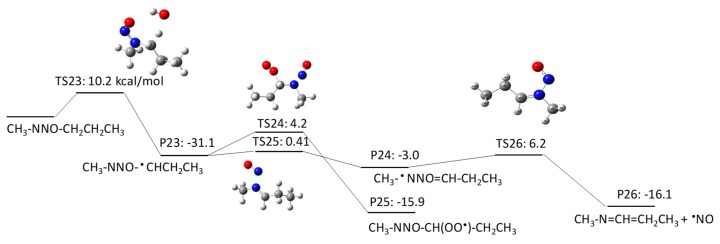
Free energy profile for pathway 3–1 of the HO^•^-induced reaction pathways for NMBA via H abstraction from a C–H bond of the –CH_2_– functional group adjacent to the N–NO functional group. The numbers (kcal/mol) are the free energy of activation for the TS and free energy of reaction for the P relative to the corresponding reactant.

**Figure 8:**
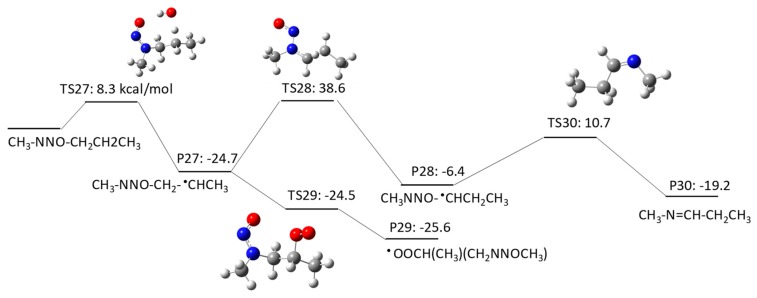
Free energy profile for pathway 3–2 of the HO^•^-induced reaction pathways for NMBA via H abstraction from a C–H bond of the -CH_2_ functional group adjacent to the –CH_2_– functional groups on both sides. The numbers (kcal/mol) are the free energy of activation for the TS and free energy of reaction for the P relative to the corresponding reactant.

**Figure 9:**
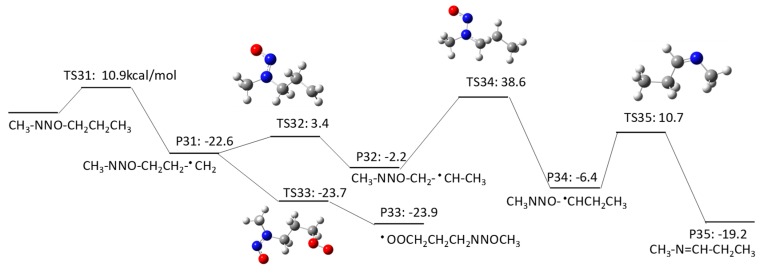
Free energy profile for pathway 3–3 of the HO^•^-induced reaction pathways for NMBA via H abstraction from a C–H bond of the terminal CH_3_ functional group in a butyl chain. The numbers (kcal/mol) are the free energy of activation for the TS and free energy of reaction for the P relative to the corresponding reactant.

**Figure 10:**
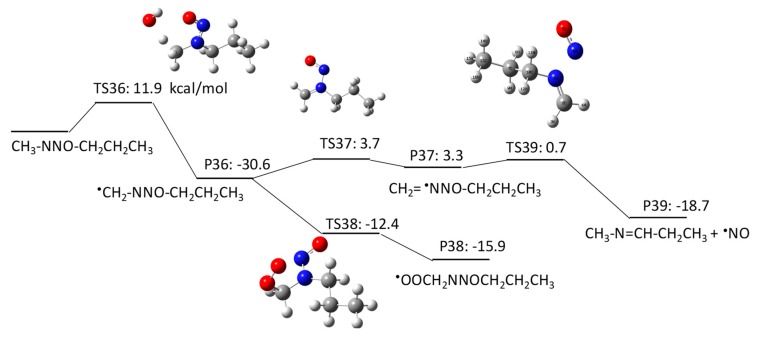
Free energy profile for pathway 3–4 of the HO^•^-induced reaction pathways for NMBA via H abstraction from a C–H bond of the terminal CH_3_ functional group adjacent to the N–NO functional group. The numbers (kcal/mol) are the free energy of activation for the TS and free energy of reaction for the P relative to the corresponding reactant.

NMBA has four potential H abstraction sites from C–H bonds by HO^•^: (1) a C–H bond of the –CH_2_– functional group adjacent to the N–NO functional group by pathway 3–1; (2) a C–H bond of the –CH_2_ functional group adjacent to the –CH_2_– functional groups on both sides by pathway 3–2; (3) a C–H bond of the terminal CH_3_ functional group in a butyl chain by pathway 3–3; and (4) a C–H bond of the terminal CH_3_ functional group that is adjacent to the N–NO functional group by pathway 3–4. Figure 7, Figure 8, Figure 9 and Figure 10 show the free energy profiles per reaction coordinate for each pathway. The calculated $\Delta G_{\mathrm{aq},\mathrm{calc}}^{\mathrm{act}}$ values are 10.2 kcal/mol for pathway 3–1, 8.3 kcal/mol for pathway 3–2, 10.9 kcal/mol for pathway 3–3, and 11.9 kcal/mol for pathway 3–4. The smaller $\Delta G_{\mathrm{aq},\mathrm{calc}}^{\mathrm{act}}$ value for pathway 3–2 compared with those for NDMA and NDEA shows consistent reactivity with the experimentally obtained rate constants: 10^9^ M^−1^s^−1^ for *N*-nitrosobutylamine, 4.3 × 10^8^ M^−1^s^−1^ for NDMA and 4.95 × 10^8^ M^−1^s^−1^ for NMEA [8]. The initial H abstraction reactions for all of the pathways are thermodynamically favorable. 

Interestingly, we observed distinctive differences in the reactivity of molecular oxygen addition to different C-centered radicals for NMBA. The initial H abstraction from different C–H bonds in NMBA produced CH_3_NNO^•^CHCH_2_CH_3_ by pathway 3–1, CH_3_NNOCH_2_^•^CHCH_3_ by pathway 3–2, CH_3_NNO(CH_2_)_2_^•^CH_2_ by pathway 3–3, and ^•^CH_2_NNO(CH_2_)_2_CH_3_ by pathway 3–4. While molecular oxygen addition to CH_3_NNO^•^CHCH_2_CH_3_ and ^•^CH_2_NNO(CH_2_)_2_CH_3_ have larger $\Delta G_{\mathrm{aq},\mathrm{calc}}^{\mathrm{act}}$ values of 4.2 kcal/mol and −12.4 kcal/mol, the $\Delta G_{\mathrm{aq},\mathrm{calc}}^{\mathrm{act}}$ values for CH_3_NNOCH_2_^•^CHCH_3_ (−25.6 kcal/mol) and CH_3_NNO(CH_2_)_2_^•^CH_2_ (−23.9 kcal/mol) are very similar to those that were observed for typical molecular oxygen addition to C-centered radicals. Thus, the alkyl side chain affects the stability of the C-centered radicals and their subsequent reactivity. The significantly slower reaction of molecular oxygen addition to the C-centered radicals produced from NDMA and NMEA may be due to the delocalization of the radical spin density from the formed C-centered radicals onto the N–NO bond(s). This radical delocalization occurs only when a terminal ^•^CH_2_ is adjacent to N–NO or ^•^CH_2_ neighbors the N–NO functional group. When the alkyl chain contains three CH_2_ functional groups, the ^•^CH_2_ three positions away from the N–NO functional group does not seem to contribute to the radical delocalization. Thus, the molecular oxygen adds to the C-centered radical without being affected by the delocalization. The different extent of radical delocalization can also explain the lower degradation efficiencies that were observed for NDMA and NEMA (approximately 80~85% degradation efficiency) as compared with nitrosodibutylamine (100% degradation efficiency) [8].

To investigate the effect of the location of the C-centered radical on the occurrence of radical delocalization, we calculated the $\Delta G_{\mathrm{aq},\mathrm{calc}}^{\mathrm{act}}$ values for radical transfer from a C-centered radical to a neighboring *C*-/*N*-centered radical. For example, CH_3_NNO^•^CHCH_2_CH_3_ undergoes radical transfer from a carbon to the amine nitrogen to produce CH_3_^•^NNO=CHCH_2_CH_3_. This reaction has a $\Delta G_{\mathrm{aq},\mathrm{calc}}^{\mathrm{act}}$ of 0.41 kcal/mol, which indicates a low barrier for this radical delocalization (pathway 3–1). Similarly, ^•^CH_2_NNO(CH_2_)_2_CH_3_ requires 3.7 kcal/mol to produce CH_2_=^•^NNO=CHCH_2_CH_3_ (pathway 3–4). In contrast, CH_3_NNOCH_2_^•^CHCH_3_ requires a $\Delta G_{\mathrm{aq},\mathrm{calc}}^{\mathrm{act}}$ of 38.6 kcal/mol to produce CH_3_NNO^•^CHCH_2_CH_3_ (pathway 3–2). A similar significantly larger $\Delta G_{\mathrm{aq},\mathrm{calc}}^{\mathrm{act}}$ value of 40.0 kcal/mol was also observed for the radical transfer reaction from ^•^CH_2_CH_2_NNOCH_3_ to CH_3_^•^CHNNOCH_3_ via pathway 2–2. Thus, there is a significant barrier for radical transfer from the functional group neighboring the N–NO functional group to the nearest CH_2_ group. Therefore, a C-centered radical in ^•^CH_2_CH_2_NNOCH_3_ or CH_3_NNOCH_2_^•^CHCH_3_ would rather undergo molecular oxygen addition than radical transfer to produce CH_3_^•^CHNNOCH_3_ or CH_3_NNO^•^CHCH_2_CH_3_, respectively.

### 2.2. UV-Induced Degradation 

#### NDMA Degradation Pathways Induced by UV Photolysis

**Figure 11:**
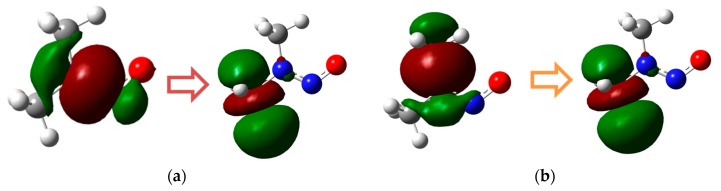
HOMO and lowest unoccupied molecular orbital (LUMO) of the π→π* (**a**) and n→π (**b**) transitions at 212 nm and 341 nm, respectively.

NDMA absorbs photons at a wavelength of 228 nm with a molar absorptivity of 7380 M^−1^cm^−1^ and quantum yield of 0.13 at pH 7 [7]. At a wavelength of 253.7 nm, where a typical low-pressure UV lamp emits photons, the molar absorptivity was reported to be 1650 M^−1^s^−1^, and the quantum yield was 0.24 at pH 7 [7]. Another smaller peak is observed at approximately 350 nm. Our TD-DFT calculation obtained one major and one minor peak at 212 nm and 341 nm, respectively. The molecular orbitals that were responsible for the π→π* and n→π transitions at 212 nm and 341 nm are shown in Figure 11. At 212 nm, the N–N bond comprises the highest occupied molecular orbital (HOMO), whereas the C–N bond comprises the HOMO at 341 nm. This analysis indicates that the N–N bond is susceptible breakage under photolysis with a low-pressure UV lamp. This finding is consistent with the experimental findings that were reported in the previous literature.

**Figure 12:**
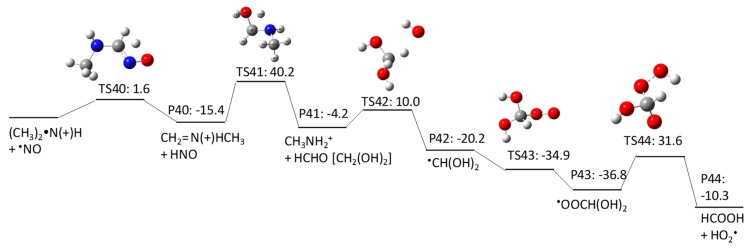
Free energy profile for pathway 4–1 of the HO^•^-induced reaction pathways for NDMA photolysis.

**Figure 13:**
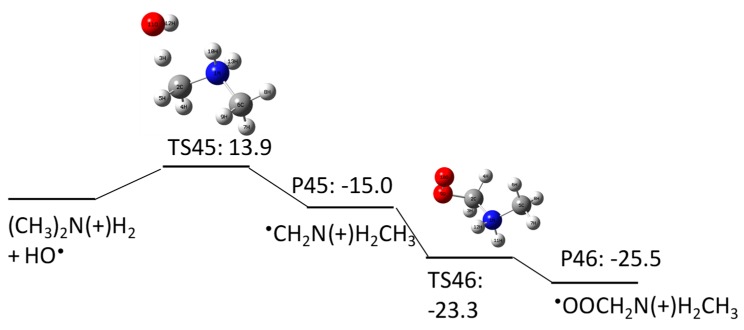
Free energy profile for pathway 4–2 of the HO^•^-induced reaction pathways for NDMA photolysis.

**Figure 14:**
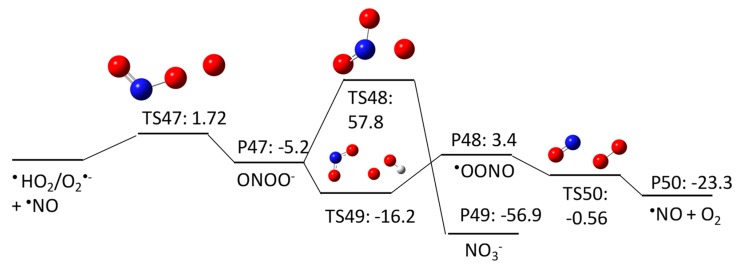
Free energy profile for pathway 4–3 of the HO^•^-induced reaction pathways for NDMA photolysis.

The UV photolysis-induced NDMA degradation pathways were extensively studied [15,16]. According to their studies, NDMA undergoes three major degradation pathways induced by UV photolysis: (1) formation of an aminium radical [(CH_3_)_2_^•^N(+)H] and nitric oxide (^•^NO) resulting from homolytic cleavage of the N–N bond (pathway 4–1 in Figure 12); (2) formation of dimethylamine [(CH_3_)_2_NH_2_^+^] and nitrous acid (HNO_2_) resulting from heterolytic photocleavage of the N–N bond facilitated by a water molecule (pathway 4–2 in Figure 13); and (3) formation of *N*-methylidenemethylamine [(CH_2_=N(+)HCH_3_], ^•^NO, and a superoxide anion radical (^•^O_2_^−^) in the presence of dissolved oxygen (i.e., triplet state of ^3^O_2_) (pathway 4–3 in Figure 14).

The products of (CH_3_)_2_^•^N(+)H and ^•^NO in pathway 4–1 react in a solvent cage to produce *N*-methylidenemethylamine [(CH_2_=N(+)HCH_3_] and nitroxyl (HNO). Our calculation obtained a $\Delta G_{\mathrm{aq},\mathrm{calc}}^{\mathrm{act}}$ of 1.6 kcal/mol for this reaction. Then, *N*-methylidenemethylamine undergoes rapid hydrolysis to produce methylamine (CH_3_NH_2_^+^) and formaldehyde (HCHO). A total of 99% of the HCHO is hydrolyzed to form a germinal diol in the aqueous phase [30]; therefore, the hydrated form of HCHO (i.e., CH_2_(OH)_2_) exists in the aqueous phase. CH_2_(OH)_2_ reacts with HO^•^ via H abstraction to produce ^•^CH(OH)_2_ with a $\Delta G_{\mathrm{aq},\mathrm{calc}}^{\mathrm{act}}$ of 10.0 kcal/mol. As was examined in the HO^•^-induced pathways, this C-centered radical reacts with molecular oxygen to produce a peroxyl radical (i.e., ^•^OOCH(OH)_2_) ($\Delta G_{\mathrm{aq},\mathrm{calc}}^{\mathrm{act}}$ of −34.9 kcal/mol). The peroxyl radical undergoes uni/bimolecular decay to produce stable lower-molecular-weight products [31]. When ^•^OOCH(OH)_2_ undergoes unimolecular decay (i.e., HO_2_^•^ elimination), formic acid (HCOOH) is produced ($\Delta G_{\mathrm{aq},\mathrm{calc}}^{\mathrm{act}}$ of 31.6 kcal/mol), which has been experimentally observed [32].

One of the C–H bonds in the methyl group of the dimethylamine produced in pathway 4–2 undergoes H abstraction by HO^•^ to produce a C-centered radical with a $\Delta G_{\mathrm{aq},\mathrm{calc}}^{\mathrm{act}}$ of 13.9 kcal/mol. Molecular oxygen adds to the C-centered radical to produce a peroxyl radical with a $\Delta G_{\mathrm{aq},\mathrm{calc}}^{\mathrm{act}}$ of −15.0 kcal/mol, and the peroxyl radical undergoes subsequent uni/bimolecular decay.

The products of ^•^NO and ^•^O_2_^-^ from pathway 4–3 react in a solvent cage to produce peroxynitrite (ONOO^−^) with a $\Delta G_{\mathrm{aq},\mathrm{calc}}^{\mathrm{act}}$ of 1.72 kcal/mol. The rate constant for this reaction was determined to be (4.3 − 7.6) × 10^9^ M^−1^s^−1^ [32,33,34]. Then, ONOO^−^ undergoes rearrangement with a $\Delta G_{\mathrm{aq},\mathrm{calc}}^{\mathrm{act}}$ of 57.8 kcal/mol to produce a nitrate ion (NO_3_^−^). This rearrangement was proposed as isomerization by Anbar and Taube (1954) [35]. ONOO^−^ also reacts with HO_2_^•^/O_2_^•−^ via single electron transfer to produce an ^•^OONO radical. Our calculation indicates that this reaction is barrierless, with a $\Delta G_{\mathrm{aq},\mathrm{calc}}^{\mathrm{act}}$ of −16.2 kcal/mol, but the reaction is not thermodynamically favorable ($\Delta G_{\mathrm{aq},\mathrm{calc}}^{\mathrm{react}}$ of 3.4 kcal/mol). Finally, the ^•^OONO radical undergoes cleavage with a $\Delta G_{\mathrm{aq},\mathrm{calc}}^{\mathrm{react}}$ of −0.56 kcal/mol to produce ^•^NO. 

**Figure 15:**
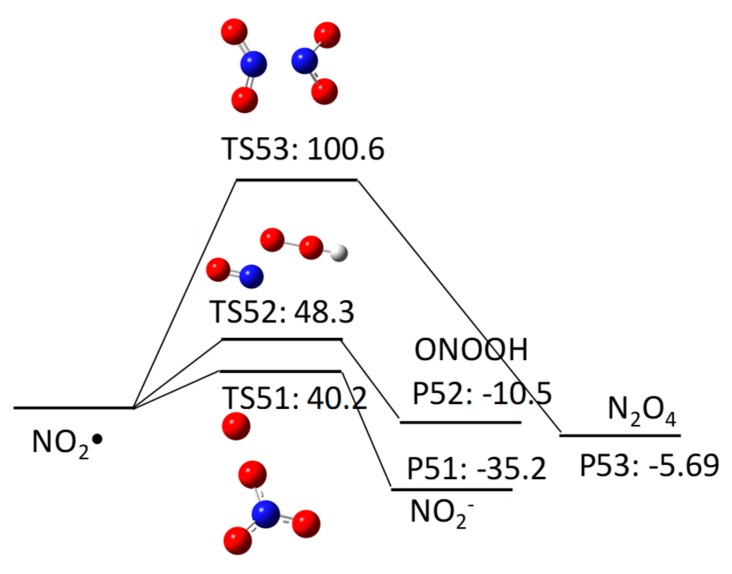
Free energy profile for the reaction of NO_2_• with HO^•^, O_2_^•−^, and NO_2_^•^.

When nitrate undergoes UV photolysis, a nitrite ion (NO_2_^−^) and NO_2_^•^ are produced. Then, NO_2_^•^ reacts with HO^•^, O_2_^•−^, or NO_2_^•^ with a $\Delta G_{\mathrm{aq},\mathrm{calc}}^{\mathrm{react}}$ of 48.3 kcal/mol, 40.2 kcal/mol, or 100.6 kcal/mol to produce ONOOH, NO_2_^-^/NO_3_^−^, or N_2_O_4_, respectively (Figure 15). Although the disproportionation of NO_2_^•^ has the largest free energy barrier, the reaction product, N_2_O_4_, undergoes hydrolysis to produce NO_3_^−^ and NO_2_^−^.

### 2.3. Environmental Implication and Future Study 

Nitrosamines, and NDMA in particular, are extremely potent carcinogenic contaminants in water. The concentration at which NDMA shows potent carcinogenicity is extremely low (0.7 ng/L) [1]. Experimentally investigating the ng/L fate of many chemical contaminants during water treatment processes is time consuming and expensive. Our computational study highlights the usefulness of QM calculations to reveal the elementary reaction pathways that are embedded in the overall reaction pathways that are typically identified by analytical techniques. This technique becomes more useful when the contaminant concentrations are below the analytical detection limit. 

Once the elementary reaction pathways are identified, the reaction rate constants should be determined or predicted to calculate the reaction rate of each molecule or species involved in each elementary reaction step. By combining the elementary reaction pathways and the reaction rate constants, one can predict the time-dependent concentration profiles of a target chemical contaminant and its transformation products. This elementary-reaction-based kinetic model could be used as an initial screening tool for many potentially toxic chemical contaminants to estimate the fate of degradation pathways. Our efforts towards the development of such elementary-reaction-based kinetic model are underway.

## 3. Materials and Methods

All of the QM calculations were performed with the Gaussian 09 revision D.02 program [36] using the Michigan Tech high-performance cluster “Superior” and homemade LINUX workstations. The M06-2X/cc-pVDZ [37] was used to optimize the electronic structures and calculate the frequencies in both the gas and aqueous phase for the HO^•^-induced reaction pathways with NDMA, NMEA, and NMBA. The UV-induced reaction pathways with NDMA was calculated with the Gaussian-4 theory (G4) [38]. The aqueous-phase structures and frequencies were obtained using an implicit polarizable continuum model [universal solvation model (SMD)] [39]. Previously, we verified the combination of M06-2X/cc-pVDZ or G4 with the SMD model by successfully applying it to other aqueous-phase radical-involved reactions [27,28]. Theoretically calculated absorption spectra were obtained from a TD-DFT analysis [40,41] of the optimized aqueous-phase structure of NDMA at the level of M06-2X/cc-pVDZ with the SMD solvation model. To investigate the contributions from molecular orbitals to the peak of the spectra, molecular orbitals were determined using a natural population analysis at the level of M06-2X/cc-pVQZ with the SMD solvation model. The detailed calculation procedures for the transition state search, the aqueous-phase free energies of activation and reaction, and the associated computational methods are found in previous reports [29].

## Supplementary Materials

Supplementary materials are available on line.

## List of Symbols and Abbreviations

AOPs	advanced oxidation processes
CH_2_=N(+)HCH_3_	*N*-methylidenemethylamine
CH_3_NHCH_3_	methyl diamine
DFT	density functional theory
$\Delta G_{\mathrm{aq},\mathrm{calc}}^{\mathrm{act}}$	theoretically calculated aqueous phase free energy of activation
$\Delta G_{\mathrm{aq},\mathrm{calc}}^{\mathrm{react}}$	theoretically calculated aqueous phase free energy of reaction
G4	Gaussian-4 theory
HCHO	formaldehyde
HCOOH	formic acid
HNO_2_	nitrous acid
HO^•^	hydroxyl radicals
HOMO	highest occupied molecular orbital
NF	nanofiltration
NDEA	*N*-nitrosodiethylamine
NDMA	*N*-nitrosodimethylamine
NMEA	*N*-nitrosomethylethylamine
NO^•^	nitric oxide
NO_3_^−^	nitrate ion
NO_2_^−^	nitrite ion
ONOO^−^	peroxynitrite
^•^O_2_^−^	superoxide anion radical
QM	quantum mechanical
RO	reverse osmosis
SMD	universal solvation model
TD-DFT	time-dependent density functional theory
TS	transition state
UV	ultraviolet
