# Supplementary material for: Mechanistic Insight into the Degradation of Nitrosamines via Aqueous-Phase UV Photolysis or a UV-Based Advanced Oxidation Process: Quantum Mechanical Calculations
Source: Molecules. 2018 Feb 28;23(3):539. doi: 10.3390/molecules23030539 (PMC6017648; doi:10.3390/molecules23030539)
Supplement: Supplementary file 1 [file molecules-23-00539-s001.pdf]

molecules

MDPI

Article

Mechanistic Insight into the Degradation of Nitrosamines in Aqueous-phase UV or UV-based Advanced Oxidation System: Quantum Mechanical Calculations

Supplemental Materials

Daisuke Minakata [1,]* and Erica Coscarelli[1]

[1] Department of Civil and Environmental Engineering, Michigan Technological University,1400 Townsend Drive, Houghton, MI, 49931; mkhare@mtu.edu

* Correspondence: dminakat@mtu.edu; Tel.: +1-906-487-1830

Academic Editor: name
Received: date; Accepted: date; Published: date

Pathway 1-1

$(CH_3)_2NNO$

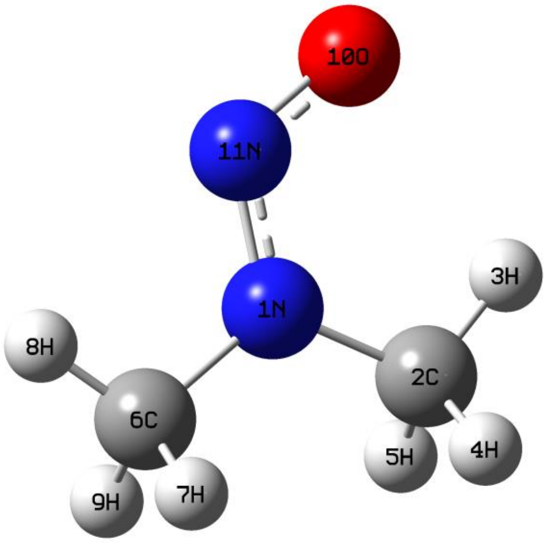

Row	Highlight	Display	Tag	Symbol	NA	NB	NC	Bond	Angle	Dihedral
1	No	Show	1	N						
2	No	Show	2	C	1			1.4568287		
3	No	Show	3	H	2	1		1.0876413	108.7050894	
4	No	Show	4	H	2	1	3	1.0937024	109.4491937	120.3865004
5	No	Show	5	H	2	1	3	1.0937082	109.4557860	-120.3875549
6	No	Show	6	C	1	2	3	1.4524329	119.3236651	-179.9498121
7	No	Show	7	H	6	1	2	1.0941437	110.0995030	59.9314814
8	No	Show	8	H	6	1	2	1.0900064	108.4145188	179.9962015
9	No	Show	9	H	6	1	2	1.0941440	110.1008003	-59.9373046
10	No	Show	10	O	1	6	2	2.1732633	147.3486737	179.9851117
11	No	Show	11	N	10	1	6	1.2400000	32.6670507	0.0222369

TS1

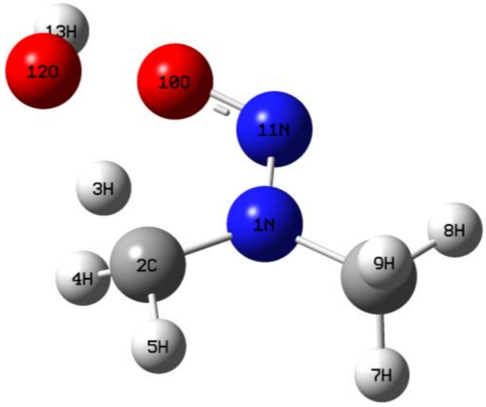

Row	Highlight	Display	Tag	Symbol	NA	NB	NC	Bond	Angle	Dihedral
1	No	Show	1	N						
2	No	Show	2	C	1			1.4298033		
3	No	Show	3	H	2	1		1.1660733	109.4228963	
4	No	Show	4	H	2	1	3	1.0909529	111.5359651	115.6655653
5	No	Show	5	H	2	1	4	1.0884771	110.1028677	126.1736837
6	No	Show	6	C	1	2	5	1.4562684	121.5805093	-9.4138368
7	No	Show	7	H	6	1	2	1.0922003	109.8916567	60.9850784
8	No	Show	8	H	6	1	2	1.0896006	108.0781735	-178.9372843
9	No	Show	9	H	6	1	2	1.0935028	109.8863769	-59.1590961
10	No	Show	10	O	1	2	6	2.1550014	89.6243717	-173.6728086

11	No	Show	11	N	10	1	2	1.2402139	33.7976533	179.2285934
12	No	Show	12	O	2	1	11	2.6067234	105.6142444	-53.2861734
13	No	Show	13	H	12	2	1	0.9753305	85.9049879	52.1792851

P1 (CH3-NNO-•CH2)

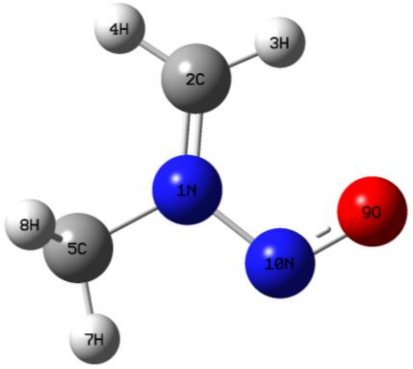

Row	Highlight	Display	Tag	Symbol	NA	NB	NC	Bond	Angle	Dihedral
1	No	Show	1	N						
2	No	Show	2	C	1			1.3161424		
3	No	Show	3	H	2	1		1.0798367	117.9071895	
4	No	Show	4	H	2	1	3	1.0800741	118.7380394	179.9887752
5	No	Show	5	C	1	2	3	1.4621912	122.5540303	-179.9975377
6	No	Show	6	H	5	1	2	1.0913285	109.7549479	60.4428595
7	No	Show	7	H	5	1	2	1.0891590	107.6137062	-179.8475651
8	No	Show	8	H	5	1	2	1.0913067	109.7485366	-60.1370326
9	No	Show	9	O	1	2	5	2.2021350	92.0916085	179.9890536
10	No	Show	10	N	9	1	2	1.2493087	35.8841163	-179.9990601

TS2

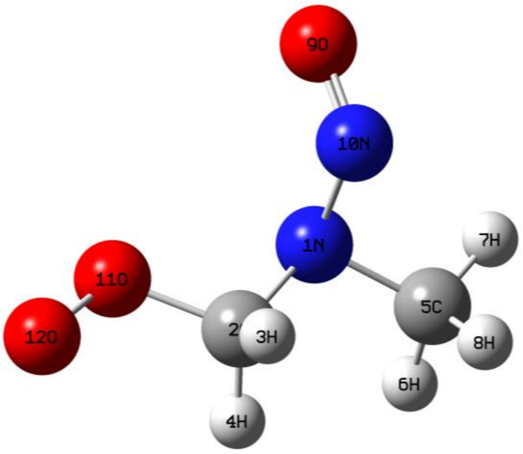

Row	Highlight	Display	Tag	Symbol	NA	NB	NC	Bond	Angle	Dihedral
1	No	Show	1	N						
2	No	Show	2	C	1			1.4375543		
3	No	Show	3	H	2	1		1.0965087	115.0250750	
4	No	Show	4	H	2	1	3	1.0910395	110.1339606	-126.0380212
5	No	Show	5	C	1	2	4	1.4781468	109.8952905	-46.4200639
6	No	Show	6	H	5	1	2	1.0916899	107.9129635	65.7794032
7	No	Show	7	H	5	1	2	1.0911626	109.0170804	-176.4215878
8	No	Show	8	H	5	1	2	1.0973078	112.9119137	-54.9362602
9	No	Show	9	O	1	2	5	2.2447130	116.7707579	-139.5132587
10	No	Show	10	N	9	1	2	1.1771453	39.2319903	70.6274352
11	No	Show	11	O	2	1	5	1.4691096	106.4713511	-162.6805357
12	No	Show	12	O	11	2	1	1.3098559	111.0640344	-169.0699712

TS3

P2

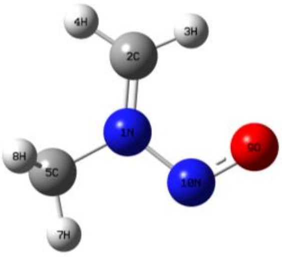

Row	Highlight	Display	Tag	Symbol	NA	NB	NC	Bond	Angle	Dihedral
1	No	Show	1	N						
2	No	Show	2	C	1			1.3161424		
3	No	Show	3	H	2	1		1.0798367	117.9071895	
4	No	Show	4	H	2	1	3	1.0800741	118.7380394	179.9887752
5	No	Show	5	C	1	2	3	1.4621912	122.5540303	-179.9975377
6	No	Show	6	H	5	1	2	1.0913285	109.7549479	60.4428595
7	No	Show	7	H	5	1	2	1.0891590	107.6137062	-179.8475651
8	No	Show	8	H	5	1	2	1.0913067	109.7485366	-60.1370326
9	No	Show	9	O	1	2	5	2.2021350	92.0916085	179.9890536
10	No	Show	10	N	9	1	2	1.2493087	35.8841163	-179.9990601

P3

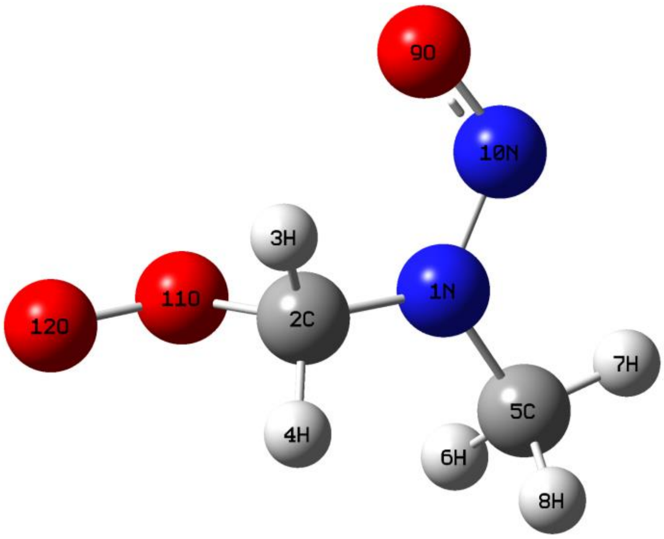

Row	Highlight	Display	Tag	Symbol	NA	NB	NC	Bond	Angle	Dihedral
1	No	Show	1	N						
2	No	Show	2	C	1			1.4260258		
3	No	Show	3	H	2	1		1.0882082	110.8830670	
4	No	Show	4	H	2	1	3	1.0884305	110.4654214	-125.5621054
5	No	Show	5	C	1	2	3	1.4583876	121.1483516	154.6717579
6	No	Show	6	H	5	1	2	1.0930170	110.0919261	60.1862469
7	No	Show	7	H	5	1	2	1.0897246	107.9208956	179.7810278
8	No	Show	8	H	5	1	2	1.0925006	110.1281321	-60.4348911
9	No	Show	9	O	1	2	5	2.1682276	90.9000330	174.4682854
10	No	Show	10	N	9	1	2	1.2257030	33.7529491	179.9490209
11	No	Show	11	O	2	1	10	1.4776556	108.0580106	86.4243309
12	No	Show	12	O	11	2	1	1.3083348	110.5660529	178.4111096

TS4

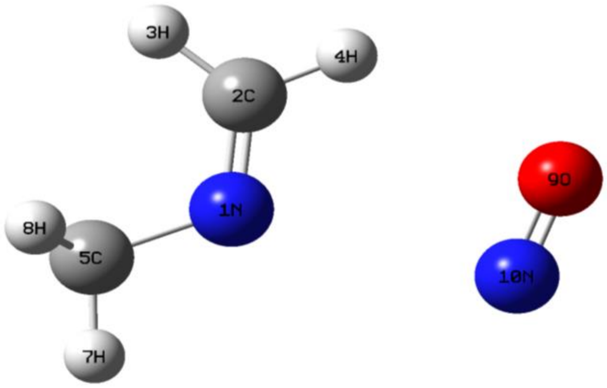

Row	Highlight	Display	Tag	Symbol	NA	NB	NC	Bond	Angle	Dihedral
1	No	Show	1	N						
2	No	Show	2	C	1			1.2683431		
3	No	Show	3	H	2	1		1.0966224	123.9880427	
4	No	Show	4	H	2	1	3	1.0909702	119.3942047	-180.0000000
5	No	Show	5	C	1	2	4	1.4662881	117.0043990	179.9990560
6	No	Show	6	H	5	1	2	1.0949899	111.0980455	60.2380318
7	No	Show	7	H	5	1	2	1.0917995	109.8235214	179.9932588
8	No	Show	8	H	5	1	2	1.0949896	111.0967873	-60.2532126
9	No	Show	9	O	2	1	5	3.2212727	79.0735081	179.9986043
10	No	Show	10	N	9	2	1	1.1544162	85.2225352	0.0043929

P4

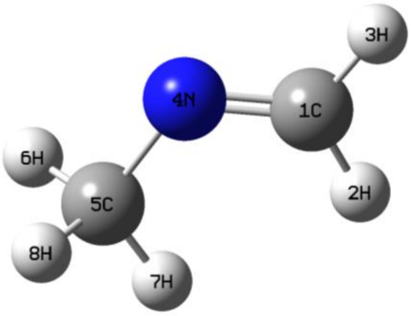

Row	Highlight	Display	Tag	Symbol	NA	NB	NC	Bond	Angle	Dihedral
1	No	Show	1	C						
2	No	Show	2	H	1			1.0968350		
3	No	Show	3	H	1	2		1.0898435	116.8631844	
4	No	Show	4	N	1	3	2	1.2715121	119.4976807	-179.9378284
5	No	Show	5	C	4	1	3	1.4548237	116.4847970	-179.9912735
6	No	Show	6	H	5	4	1	1.0947055	109.2434968	-121.8552085
7	No	Show	7	H	5	4	1	1.0978746	113.6840652	0.0102200
8	No	Show	8	H	5	4	1	1.0946601	109.2546109	121.8927213

Pathway 1-2

TS5

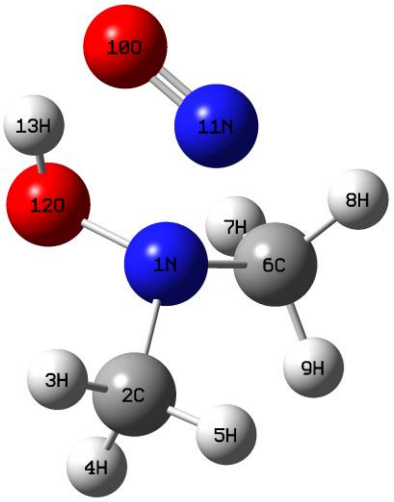

Row	Highlight	Display	Tag	Symbol	NA	NB	NC	Bond	Angle	Dihedral
1	No	Show	1	N						
2	No	Show	2	C	1			1.4569985		
3	No	Show	3	H	2	1		1.0914789	108.9222629	
4	No	Show	4	H	2	1	3	1.0975779	111.3827540	121.0480784
5	No	Show	5	H	2	1	3	1.0924138	108.3773128	-118.6913298
6	No	Show	6	C	1	2	3	1.4693886	115.9300548	175.8026202
7	No	Show	7	H	6	1	2	1.0908424	109.1200646	121.6449344
8	No	Show	8	H	6	1	2	1.0938829	109.7604942	-118.6918474
9	No	Show	9	H	6	1	2	1.0927972	110.4540806	1.7982376
10	No	Show	10	O	1	2	6	2.6316959	116.9659899	-150.1144784
11	No	Show	11	N	10	1	2	1.1929329	50.7696593	78.0715557
12	No	Show	12	O	1	2	6	1.3983760	109.9974639	127.3055187
13	No	Show	13	H	12	1	2	0.9839695	103.7954009	112.3702310

P5

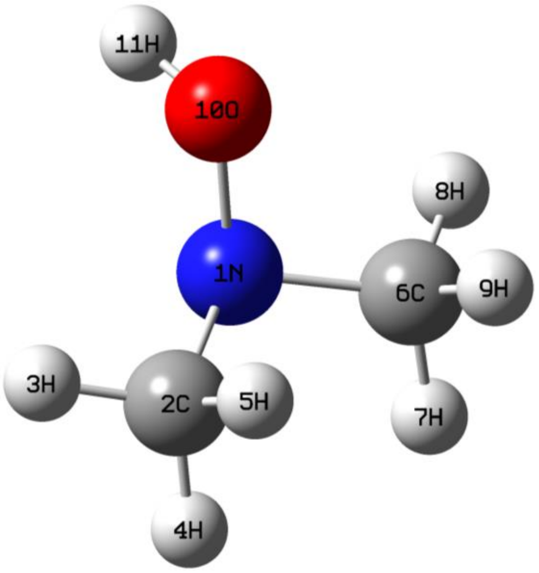

Row	Highlight	Display	Tag	Symbol	NA	NB	NC	Bond	Angle	Dihedral
1	No	Show	1	N						
2	No	Show	2	C	1			1.4601490		
3	No	Show	3	H	2	1		1.0921199	109.6344162	
4	No	Show	4	H	2	1	3	1.0933223	108.4902188	118.3758424
5	No	Show	5	H	2	1	4	1.1006068	112.4573725	120.3206899
6	No	Show	6	C	1	2	3	1.4602495	110.4708477	177.6829790
7	No	Show	7	H	6	1	2	1.0932969	108.5133527	63.9250683
8	No	Show	8	H	6	1	2	1.0921144	109.6145982	-177.6754574
9	No	Show	9	H	6	1	2	1.1006085	112.4450152	-56.4027286
10	No	Show	10	O	1	2	6	1.4576323	105.3096817	-113.1788977
11	No	Show	11	H	10	1	2	0.9650138	103.4132598	-121.4607138

TS6

P6

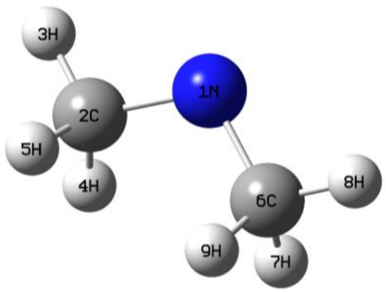

Row	Highlight	Display	Tag	Symbol	NA	NB	NC	Bond	Angle	Dihedral
1	No	Show	1	N						
2	No	Show	2	C	1			1.4399579		
3	No	Show	3	H	2	1		1.0922830	110.9335532	
4	No	Show	4	H	2	1	3	1.1004971	111.8662577	121.8097228
5	No	Show	5	H	2	1	3	1.1012208	110.6487485	-120.1778077
6	No	Show	6	C	1	2	3	1.4399579	110.6968204	-174.9022460
7	No	Show	7	H	6	1	2	1.1012208	110.6487485	64.9199463
8	No	Show	8	H	6	1	2	1.0922830	110.9335532	-174.9022460
9	No	Show	9	H	6	1	2	1.1004971	111.8662577	-53.0925232

TS7

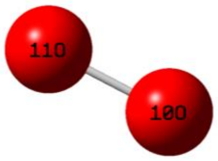

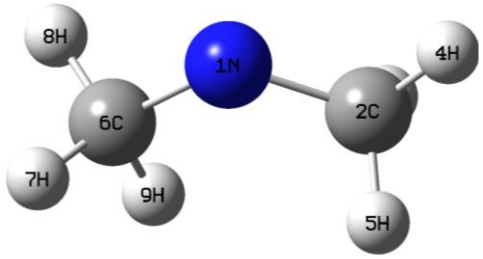

Row	Highlight	Display	Tag	Symbol	NA	NB	NC	Bond	Angle	Dihedral
1	No	Show	1	N						
2	No	Show	2	C	1			1.4601651		
3	No	Show	3	H	2	1		1.0968539	112.7808185	
4	No	Show	4	H	2	1	3	1.0914807	109.2367340	122.5086927
5	No	Show	5	H	2	1	4	1.0916196	107.3501883	117.2808560
6	No	Show	6	C	1	2	4	1.4663259	114.5264627	172.6627217
7	No	Show	7	H	6	1	2	1.0922071	110.3049893	123.4210007
8	No	Show	8	H	6	1	2	1.0917787	109.4691362	-117.8519399
9	No	Show	9	H	6	1	2	1.0927559	110.2356221	2.4662359
10	No	Show	10	O	1	2	6	1.4793148	103.1516951	-120.6554929
11	No	Show	11	O	10	1	2	1.2896223	114.6925236	162.0244429

TS8

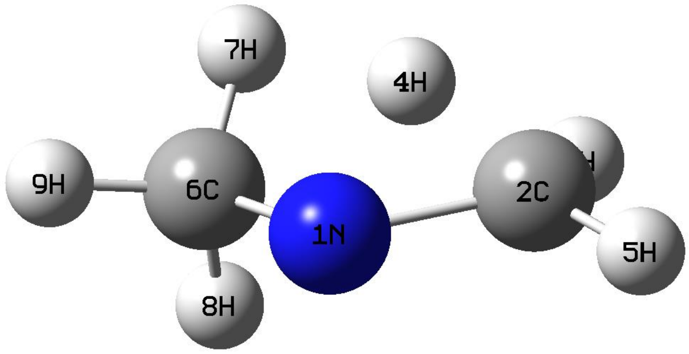

Row	Highlight	Display	Tag	Symbol	NA	NB	NC	Bond	Angle	Dihedral
1	No	Show	1	N						
2	No	Show	2	C	1			1.4451364		
3	No	Show	3	H	2	1		1.0877322	120.3124602	
4	No	Show	4	H	1	2	3	1.2241581	57.1431930	-110.6350017
5	No	Show	5	H	2	1	4	1.0846293	115.4220957	95.1725145
6	No	Show	6	C	1	2	5	1.4620514	110.7719858	-165.9813146
7	No	Show	7	H	6	1	2	1.0978134	113.0921219	-43.3215830
8	No	Show	8	H	6	1	2	1.0977636	109.6126957	76.3564551

9	No	Show	9	H	6	1	2	1.0922577	110.0836425	-164.7087497

P7

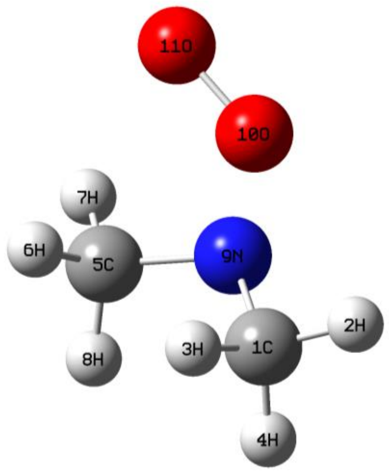

Row	Highlight	Display	Tag	Symbol	NA	NB	NC	Bond	Angle	Dihedral
1	No	Show	1	C						
2	No	Show	2	H	1			1.0914194		
3	No	Show	3	H	1	2		1.0971352	109.6696262	
4	No	Show	4	H	1	2	3	1.0913491	108.4491644	-119.4818390
5	No	Show	5	C	1	4	2	2.4420018	95.2369285	149.0911215
6	No	Show	6	H	5	1	4	1.0975448	93.6161955	117.9810016
7	No	Show	7	H	5	1	4	1.0891663	143.7237458	-111.0337953
8	No	Show	8	H	5	1	4	1.0919141	88.2109414	8.8141912
9	No	Show	9	N	5	1	4	1.4533789	33.2302644	-114.8108959
10	No	Show	10	O	9	5	1	1.4813762	110.4343568	-115.3564094
11	No	Show	11	O	10	9	5	1.2917186	115.1509932	-21.6557791

P8

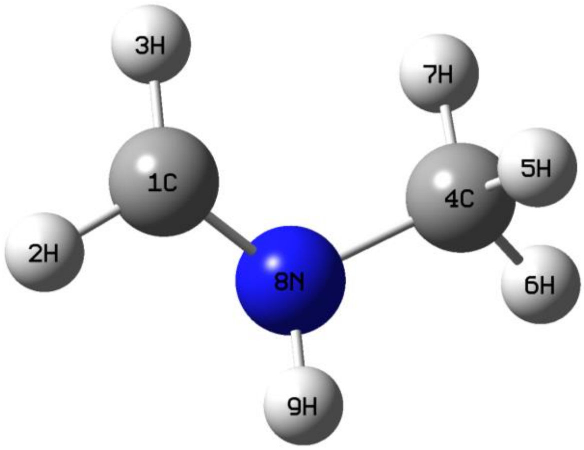

Row	Highlight	Display	Tag	Symbol	NA	NB	NC	Bond	Angle	Dihedral
1	No	Show	1	C						
2	No	Show	2	H	1			1.0853877		
3	No	Show	3	H	1	2		1.0866627	118.0786662	

4	No	Show	4	C	1	2	3	2.4371546	147.3582594	-150.5187851
5	No	Show	5	H	4	1	2	1.1000145	98.3329531	-111.1981819
6	No	Show	6	H	4	1	2	1.0926038	139.7957995	20.6808379
7	No	Show	7	H	4	1	2	1.0945397	90.8280230	140.6307712
8	No	Show	8	N	1	4	6	1.3889762	31.8191469	9.4893280
9	No	Show	9	H	8	1	4	1.0125256	113.5956378	134.8578016

Pathway 1-3

TS9

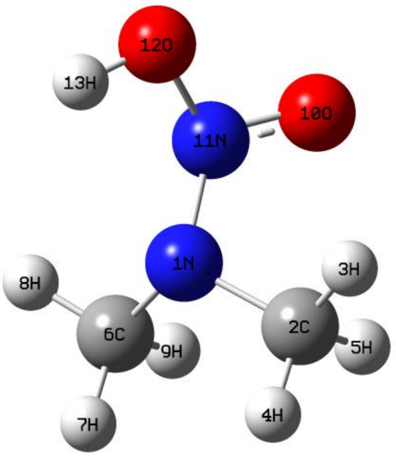

Row	Highlight	Display	Tag	Symbol	NA	NB	NC	Bond	Angle	Dihedral
1	No	Show	1	N						
2	No	Show	2	C	1			1.4680285		
3	No	Show	3	H	2	1		1.0915764	109.5382116	
4	No	Show	4	H	2	1	3	1.0918110	107.0232181	117.8465242
5	No	Show	5	H	2	1	4	1.0963427	112.9116577	119.8305269
6	No	Show	6	C	1	2	3	1.4707272	112.1777318	177.5663276
7	No	Show	7	H	6	1	2	1.0917023	107.8898100	62.5067535
8	No	Show	8	H	6	1	2	1.0916521	109.5432511	-179.2856500
9	No	Show	9	H	6	1	2	1.0984871	112.3103217	-57.5370198
10	No	Show	10	O	1	2	6	2.3437243	85.2701316	-111.1200668
11	No	Show	11	N	10	1	2	1.2449946	31.6473427	-160.6774879
12	No	Show	12	O	11	10	1	1.4236754	113.1522708	135.8730880
13	No	Show	13	H	12	11	10	0.9709777	103.8437549	-173.7998733

P9

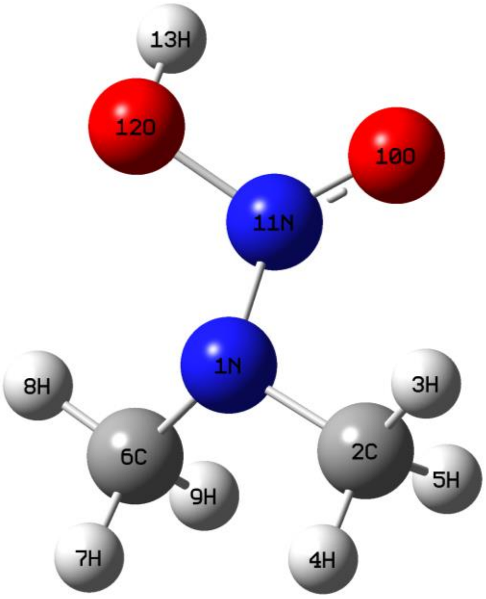

Row	Highlight	Display	Tag	Symbol	NA	NB	NC	Bond	Angle	Dihedral
1	No	Show	1	N						
2	No	Show	2	C	1			1.4635942		
3	No	Show	3	H	2	1		1.0891337	110.1167791	
4	No	Show	4	H	2	1	3	1.0908062	106.7271340	118.2954113
5	No	Show	5	H	2	1	4	1.0977723	112.5962273	119.3614203
6	No	Show	6	C	1	2	3	1.4700875	112.4762731	-175.1557087
7	No	Show	7	H	6	1	2	1.0909071	107.8623692	64.2651032
8	No	Show	8	H	6	1	2	1.0889918	109.5455174	-177.5170281
9	No	Show	9	H	6	1	2	1.0975324	112.0170387	-55.7861895
10	No	Show	10	O	1	2	6	2.3153311	85.5526209	-135.4458246
11	No	Show	11	N	10	1	2	1.2495681	32.2559248	153.1312055
12	No	Show	12	O	11	10	1	1.4409188	116.2207248	130.1236851
13	No	Show	13	H	12	11	10	0.9709238	102.1697911	51.8057450

P10

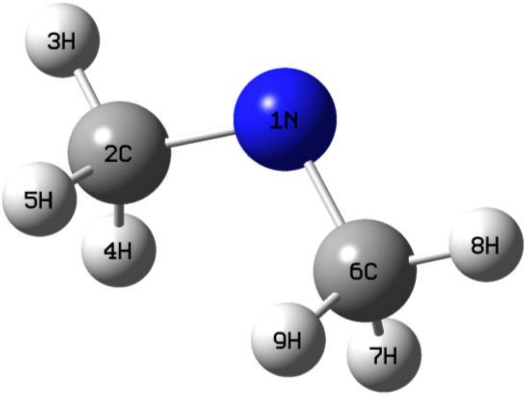

Row	Highlight	Display	Tag	Symbol	NA	NB	NC	Bond	Angle	Dihedral
1	No	Show	1	N						
2	No	Show	2	C	1			1.4399579		

3	No	Show	3	H	2	1		1.0922830	110.9335532	
4	No	Show	4	H	2	1	3	1.1004971	111.8662577	121.8097228
5	No	Show	5	H	2	1	3	1.1012208	110.6487485	-120.1778077
6	No	Show	6	C	1	2	3	1.4399579	110.6968204	-174.9022460
7	No	Show	7	H	6	1	2	1.1012208	110.6487485	64.9199463
8	No	Show	8	H	6	1	2	1.0922830	110.9335532	-174.9022460
9	No	Show	9	H	6	1	2	1.1004971	111.8662577	-53.0925232

P11

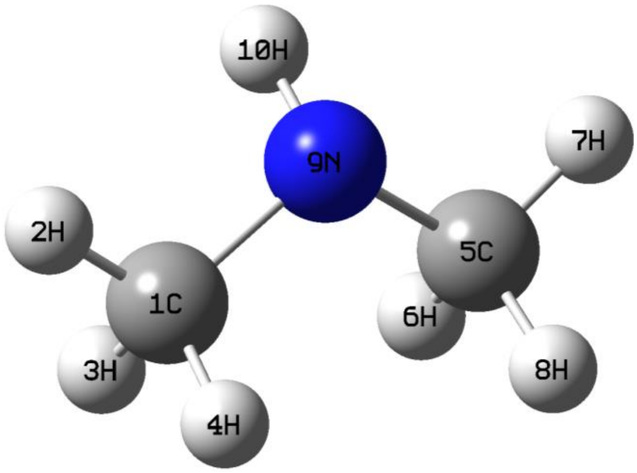

Row	Highlight	Display	Tag	Symbol	NA	NB	NC	Bond	Angle	Dihedral
1	No	Show	1	C						
2	No	Show	2	H	1			1.0936924		
3	No	Show	3	H	1	2		1.1032237	108.0525516	
4	No	Show	4	H	1	2	3	1.0956165	107.7824685	-115.6537590
5	No	Show	5	C	1	2	4	2.4095480	144.3105390	-123.7171349
6	No	Show	6	H	5	1	2	1.1032237	91.7710014	-125.0635832
7	No	Show	7	H	5	1	2	1.0936924	144.3105390	0.0000000
8	No	Show	8	H	5	1	2	1.0956165	93.4580976	127.4868983
9	No	Show	9	N	5	1	2	1.4614110	34.4732072	6.8271493
10	No	Show	10	H	9	5	1	1.0167389	107.0839348	-116.5911491

TS11

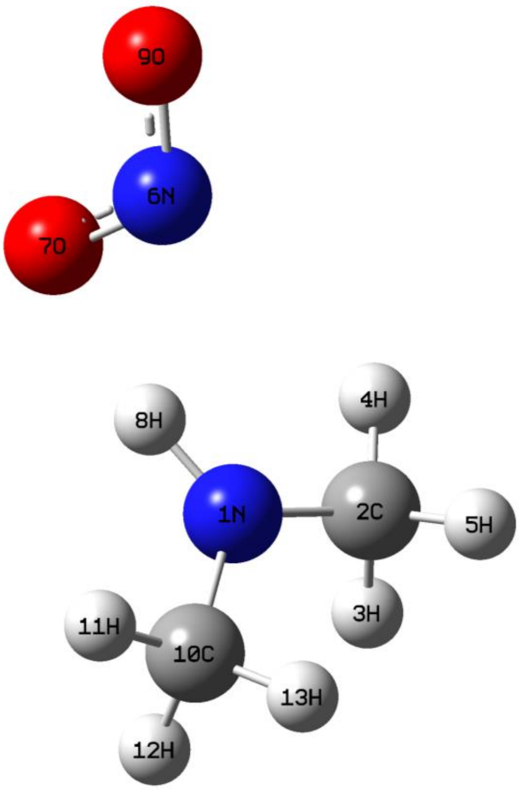

Row	Highlight	Display	Tag	Symbol	NA	NB	NC	Bond	Angle	Dihedral
1	No	Show	1	N						
2	No	Show	2	C	1			1.4318095		
3	No	Show	3	H	2	1		1.0990542	109.8184278	
4	No	Show	4	H	2	1	3	1.0888511	109.8739958	121.4017611
5	No	Show	5	H	2	1	4	1.0977322	109.7175653	121.3114068
6	No	Show	6	N	1	2	4	2.8277076	108.1400296	-30.3676204
7	No	Show	7	O	6	1	2	1.2665598	70.9499890	110.7622207
8	No	Show	8	H	1	2	6	1.0494585	118.8004486	35.7799502
9	No	Show	9	O	6	1	2	1.2339902	171.3425683	-55.3196328
10	No	Show	10	C	1	2	6	1.4321576	120.6996395	-153.7766169
11	No	Show	11	H	10	1	2	1.0895569	109.8636772	169.7214270
12	No	Show	12	H	10	1	2	1.1012890	109.5188781	-70.3416221
13	No	Show	13	H	10	1	2	1.0952168	110.0655853	47.2926346

Pathway 2-1

CH3CH2NNOCH3

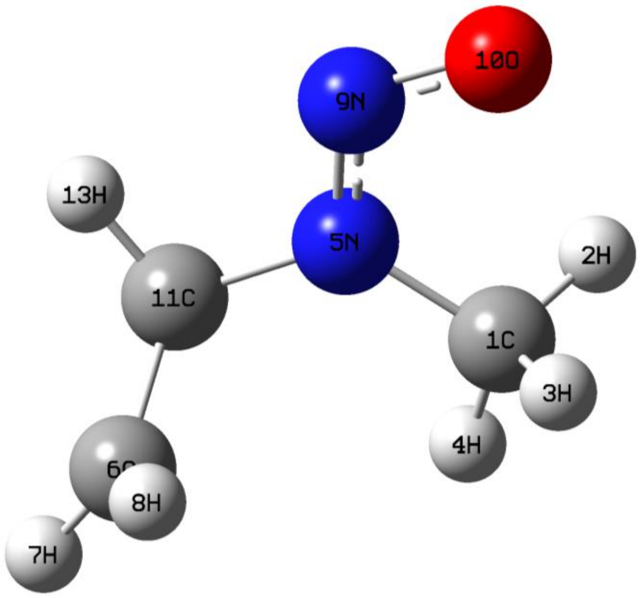

Row	Highlight	Display	Tag	Symbol	NA	NB	NC	Bond	Angle	Dihedral
1	No	Show	1	C						
2	No	Show	2	H	1			1.0930419		
3	No	Show	3	H	1	2		1.0938291	108.3762702	
4	No	Show	4	H	1	2	3	1.0886719	110.1149353	-120.1508015
5	No	Show	5	N	1	4	3	1.4583877	108.3087268	-120.7743019
6	No	Show	6	C	5	1	4	2.4619382	102.3754974	35.7210646
7	No	Show	7	H	6	5	1	1.0828968	144.7153651	-75.7663084
8	No	Show	8	H	6	5	1	1.0841443	94.0441635	85.0323235
9	No	Show	9	N	5	1	6	1.3083450	121.1636750	149.0287177
10	No	Show	10	O	9	5	1	1.2428842	114.9221064	-0.0939093
11	No	Show	11	C	5	1	9	1.4664845	121.7931482	-179.0951293
12	No	Show	12	H	11	5	1	1.0955196	108.6460740	-61.5558315
13	No	Show	13	H	11	5	1	1.0993087	105.0860072	-175.7751436

TS12

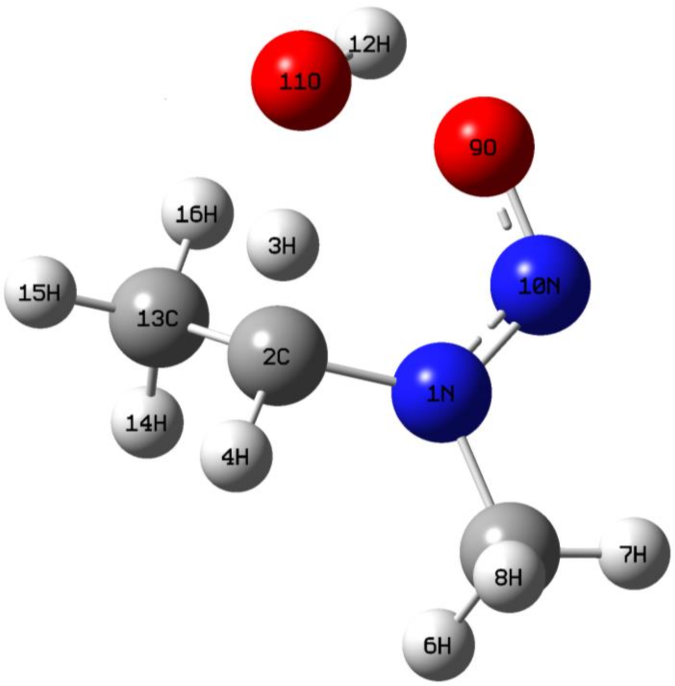

Row	Highlight	Display	Tag	Symbol	NA	NB	NC	Bond	Angle	Dihedral
1	No	Show	1	N						
2	No	Show	2	C	1			1.4480403		
3	No	Show	3	H	2	1		1.1616656	107.7076893	
4	No	Show	4	H	2	1	3	1.0911383	107.1964464	-112.5632477
5	No	Show	5	C	1	2	4	1.4576177	120.1077066	9.4511867
6	No	Show	6	H	5	1	2	1.0925526	109.9603476	60.8512768
7	No	Show	7	H	5	1	2	1.0897173	108.2400705	-179.0071954
8	No	Show	8	H	5	1	2	1.0936481	109.8404692	-59.3237705
9	No	Show	9	O	1	2	5	2.1658698	92.4518518	-175.2603261
10	No	Show	10	N	9	1	2	1.2413396	33.1592543	179.5343782
11	No	Show	11	O	2	1	10	2.6291744	104.6515041	-44.9745542
12	No	Show	12	H	11	2	1	0.9760102	86.8757675	45.4175529
13	No	Show	13	C	2	1	10	1.5219281	115.7965522	68.4232506
14	No	Show	14	H	13	2	1	1.0953200	110.0537693	61.4125225
15	No	Show	15	H	13	2	1	1.0929403	108.8858357	179.9848847
16	No	Show	16	H	13	2	1	1.0901559	111.9238865	-59.5759518

TS13

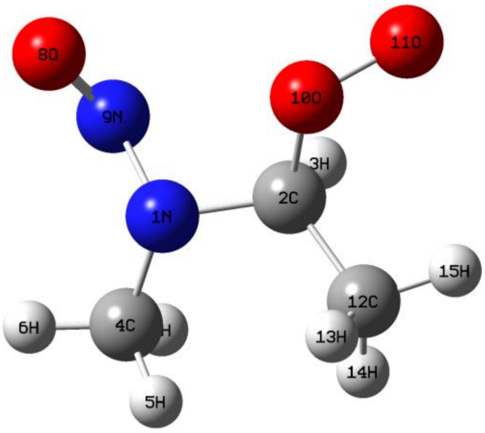

Row	Highlight	Display	Tag	Symbol	NA	NB	NC	Bond	Angle	Dihedral
1	No	Show	1	N						
2	No	Show	2	C	1			1.4483591		
3	No	Show	3	H	2	1		1.0973252	113.0493102	
4	No	Show	4	C	1	2	3	1.4766563	112.3522086	76.4717803
5	No	Show	5	H	4	1	2	1.0901335	108.6793598	70.4622551
6	No	Show	6	H	4	1	2	1.0917089	108.3841510	-172.4532360
7	No	Show	7	H	4	1	2	1.0973760	113.2566787	-51.2864462
8	No	Show	8	O	1	2	4	2.2489493	116.0443459	-138.8454849
9	No	Show	9	N	8	1	2	1.1770599	39.2450088	69.8560307
10	No	Show	10	O	2	1	4	1.4885359	102.8410054	-171.1558779
11	No	Show	11	O	10	2	1	1.3089050	111.6684114	-159.0434874
12	No	Show	12	C	2	1	4	1.5132180	115.0974438	-53.2096540
13	No	Show	13	H	12	2	1	1.0926569	111.3490728	-48.4342762
14	No	Show	14	H	12	2	1	1.0917530	110.4190229	73.4098661
15	No	Show	15	H	12	2	1	1.0925069	108.0939636	-168.4990405

TS14

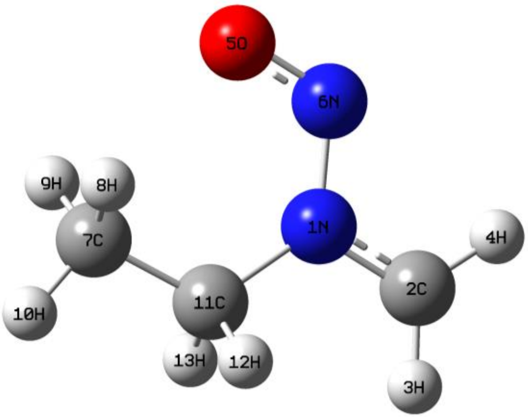

Row	Highlight	Display	Tag	Symbol	NA	NB	NC	Bond	Angle	Dihedral
1	No	Show	1	N						
2	No	Show	2	C	1			1.3335576		
3	No	Show	3	H	2	1		1.0787447	118.9218232	

4	No	Show	4	H	2	1	3	1.0803686	118.1273217	180.0000000
5	No	Show	5	O	1	2	3	2.2234578	145.8482388	180.0000000
6	No	Show	6	N	5	1	2	1.2484082	33.8175881	0.0000000
7	No	Show	7	C	1	2	6	2.6220795	146.4092694	180.0000000
8	No	Show	8	H	7	1	2	1.0897025	96.8966620	124.9248795
9	No	Show	9	H	7	1	2	1.0897025	96.8966620	-124.9248795
10	No	Show	10	H	7	1	2	1.0961768	135.6895637	0.0000000
11	No	Show	11	C	1	2	6	1.5059037	116.2221739	180.0000000
12	No	Show	12	H	11	1	2	1.0924162	104.7086855	56.4345376
13	No	Show	13	H	11	1	2	1.0924154	104.7086966	-56.4345076

TS15

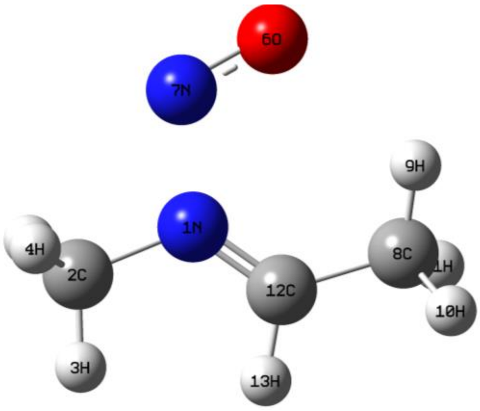

Row	Highlight	Display	Tag	Symbol	NA	NB	NC	Bond	Angle	Dihedral
1	No	Show	1	N						
2	No	Show	2	C	1			1.4554416		
3	No	Show	3	H	2	1		1.0906363	110.6283971	
4	No	Show	4	H	2	1	3	1.0922129	108.8862296	120.5554114
5	No	Show	5	H	2	1	4	1.0923102	109.4942008	118.3515533
6	No	Show	6	O	1	2	3	2.3935575	135.3306868	177.6192746
7	No	Show	7	N	6	1	2	1.2349753	38.7165750	12.8782839
8	No	Show	8	C	1	2	7	2.4873900	150.2838943	179.4879307
9	No	Show	9	H	8	1	2	1.0843239	89.6807448	-169.3420407
10	No	Show	10	H	8	1	2	1.0959089	122.5546941	-55.2997938
11	No	Show	11	H	8	1	2	1.0978213	116.9877801	78.7481827
12	No	Show	12	C	1	2	7	1.2849397	121.9577457	-178.8008398
13	No	Show	13	H	12	1	2	1.0911043	116.2177333	1.1958753

Pathway

TS16

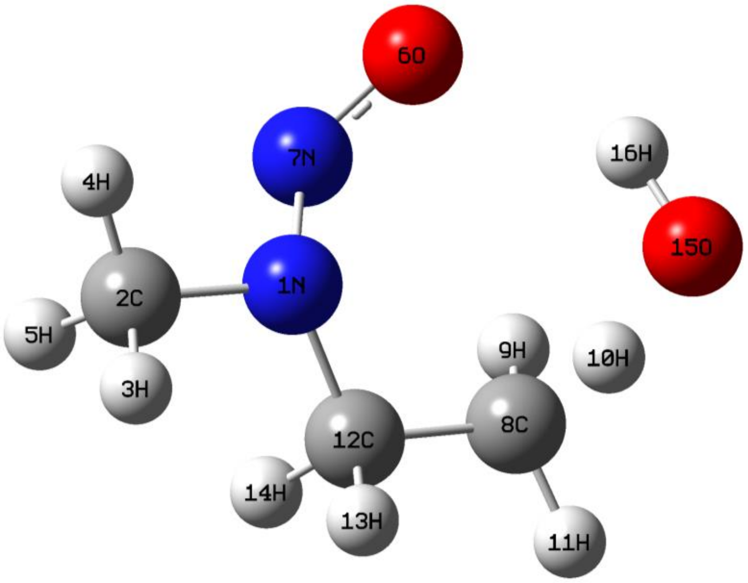

Row	Highlight	Display	Tag	Symbol	NA	NB	NC	Bond	Angle	Dihedral
1	No	Show	1	N						
2	No	Show	2	C	1			1.4649917		
3	No	Show	3	H	2	1		1.0920570	107.6504330	
4	No	Show	4	H	2	1	3	1.0906767	109.1561140	118.6358429
5	No	Show	5	H	2	1	3	1.0961524	112.1879422	-119.7010663
6	No	Show	6	O	1	2	4	2.3488200	127.4194101	-30.6376704
7	No	Show	7	N	6	1	2	1.2430704	28.9445771	-58.5528574
8	No	Show	8	C	1	7	6	2.4975521	89.1271663	-72.9402128
9	No	Show	9	H	8	1	7	1.0915134	97.5634396	-14.2283576
10	No	Show	10	H	8	1	7	1.1955920	87.9772386	90.6337019
11	No	Show	11	H	8	1	7	1.0920982	143.4884676	-156.3848999
12	No	Show	12	C	1	7	6	1.4777646	113.4424544	-96.7190806
13	No	Show	13	H	12	1	7	1.0939578	105.3876482	168.6923925
14	No	Show	14	H	12	1	7	1.1007244	109.9115365	-76.1316161
15	No	Show	15	O	8	1	7	2.5589287	82.5868282	84.9708770
16	No	Show	16	H	15	8	1	0.9747580	88.6905015	-53.1669150

TS17

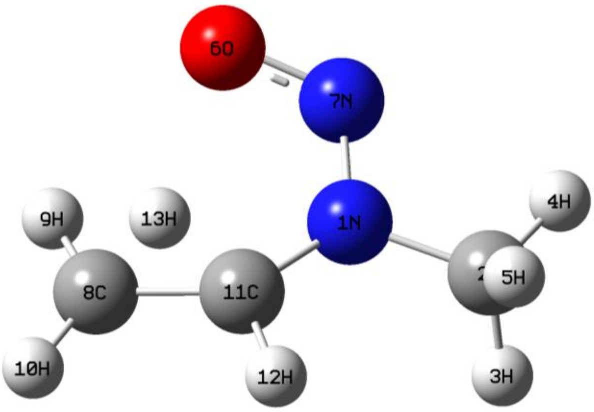

Row	Highlight	Display	Tag	Symbol	NA	NB	NC	Bond	Angle	Dihedral
1	No	Show	1	N						
2	No	Show	2	C	1			1.4620341		
3	No	Show	3	H	2	1		1.0921752	110.6263818	
4	No	Show	4	H	2	1	3	1.0892566	107.4461271	119.8394657
5	No	Show	5	H	2	1	4	1.0930933	110.3486376	119.0260063
6	No	Show	6	O	1	2	4	2.1879719	145.6948058	-13.1882701
7	No	Show	7	N	6	1	2	1.2622427	34.3913082	16.9278121
8	No	Show	8	C	1	7	6	2.5673660	102.5932002	15.6016741
9	No	Show	9	H	8	1	7	1.0751875	98.2384183	13.1331261
10	No	Show	10	H	8	1	7	1.0814570	139.8367340	-170.4765698
11	No	Show	11	C	1	7	6	1.3833155	126.2189791	2.2081798
12	No	Show	12	H	11	1	7	1.0831741	112.9048078	-168.8789150
13	No	Show	13	H	11	1	7	1.2523589	115.7584993	-41.9325129

TS18

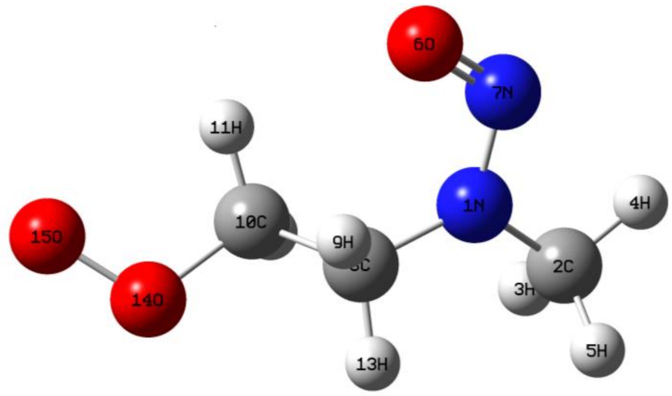

Row	Highlight	Display	Tag	Symbol	NA	NB	NC	Bond	Angle	Dihedral
1	No	Show	1	N						
2	No	Show	2	C	1			1.4485433		
3	No	Show	3	H	2	1		1.0958596	111.2226087	
4	No	Show	4	H	2	1	3	1.0908621	107.3450035	119.3820956
5	No	Show	5	H	2	1	4	1.0958514	111.0185778	119.5540348
6	No	Show	6	O	1	2	4	2.1578147	147.5529145	-0.6884636
7	No	Show	7	N	6	1	2	1.2201443	34.3246045	-0.8915772
8	No	Show	8	C	1	7	6	1.4560585	121.0502111	-2.6925460
9	No	Show	9	H	8	1	7	1.0930302	108.0761032	-38.5029839
10	No	Show	10	C	8	1	7	1.5308759	111.7388457	81.7523387
11	No	Show	11	H	10	8	1	1.0888184	112.0444544	-70.5035867
12	No	Show	12	H	10	8	1	1.0954829	111.4428384	53.7530160
13	No	Show	13	H	8	1	7	1.0928014	108.1724623	-156.9199235
14	No	Show	14	O	10	8	1	1.4529866	107.3743870	170.9059452
15	No	Show	15	O	14	10	8	1.3144352	112.3778313	133.6034651

TS19

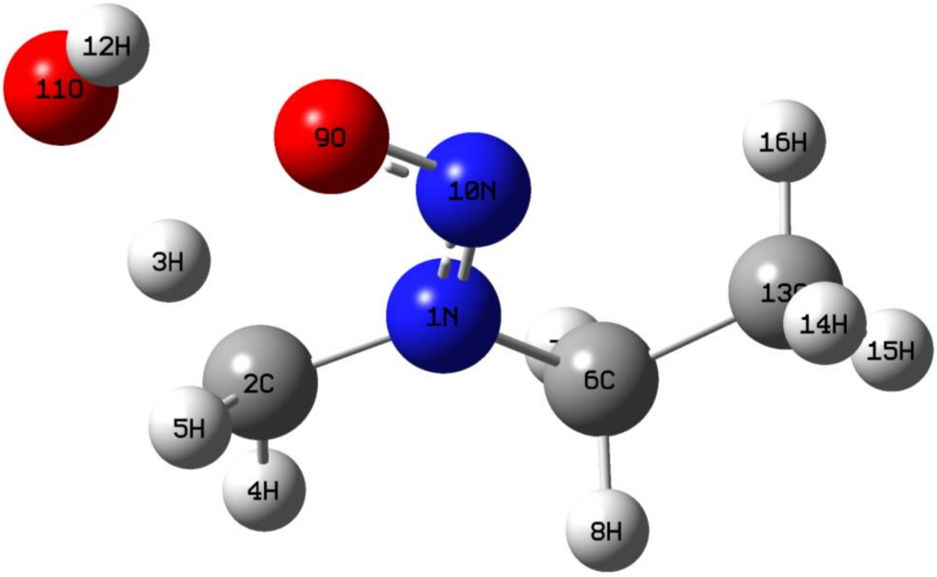

Row	Highlight	Display	Tag	Symbol	NA	NB	NC	Bond	Angle	Dihedral
1	No	Show	1	N						
2	No	Show	2	C	1			1.4300719		
3	No	Show	3	H	2	1		1.1695185	109.3662399	
4	No	Show	4	H	2	1	3	1.0883004	110.2416976	118.1157555
5	No	Show	5	H	2	1	4	1.0910063	111.7369802	126.3781161
6	No	Show	6	C	1	2	4	1.4729949	120.1463783	6.2702978
7	No	Show	7	H	6	1	2	1.0950168	106.9829757	55.4421572
8	No	Show	8	H	6	1	2	1.0942843	106.9387884	-59.0723147
9	No	Show	9	O	1	2	6	2.1546848	89.2940418	176.1419648
10	No	Show	10	N	9	1	2	1.2439177	33.6406092	179.9664902
11	No	Show	11	O	2	1	10	2.5969230	105.4639763	53.3652019
12	No	Show	12	H	11	2	1	0.9754838	85.4743703	-53.7095488
13	No	Show	13	C	6	1	10	1.5183748	114.1599288	1.9295061
14	No	Show	14	H	13	6	1	1.0924477	111.7942732	60.4346536
15	No	Show	15	H	13	6	1	1.0936271	107.7447353	179.3590583
16	No	Show	16	H	13	6	1	1.0924374	111.8564822	-61.6231326

TS20

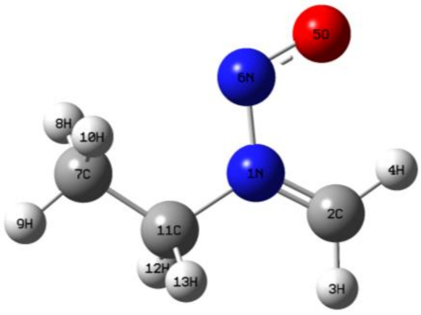

Row	Highlight	Display	Tag	Symbol	NA	NB	NC	Bond	Angle	Dihedral

1	No	Show	1	N						
2	No	Show	2	C	1			1.3198271		
3	No	Show	3	H	2	1		1.0798681	118.7371622	
4	No	Show	4	H	2	1	3	1.0790779	118.2110887	180.0000000
5	No	Show	5	O	1	2	4	2.1971428	91.4999655	-0.0007252
6	No	Show	6	N	5	1	2	1.2517924	35.6601502	180.0000000
7	No	Show	7	C	1	2	6	2.5261853	153.6887383	-179.9793137
8	No	Show	8	H	7	1	2	1.0917744	94.5373085	-125.4108780
9	No	Show	9	H	7	1	2	1.0940138	139.6542106	-0.0298870
10	No	Show	10	H	7	1	2	1.0917770	94.5334992	125.3453983
11	No	Show	11	C	1	2	6	1.4833324	120.5270406	179.9976292
12	No	Show	12	H	11	1	2	1.0927890	106.6121220	-57.4280026
13	No	Show	13	H	11	1	2	1.0927880	106.6106493	57.3920450

TS21

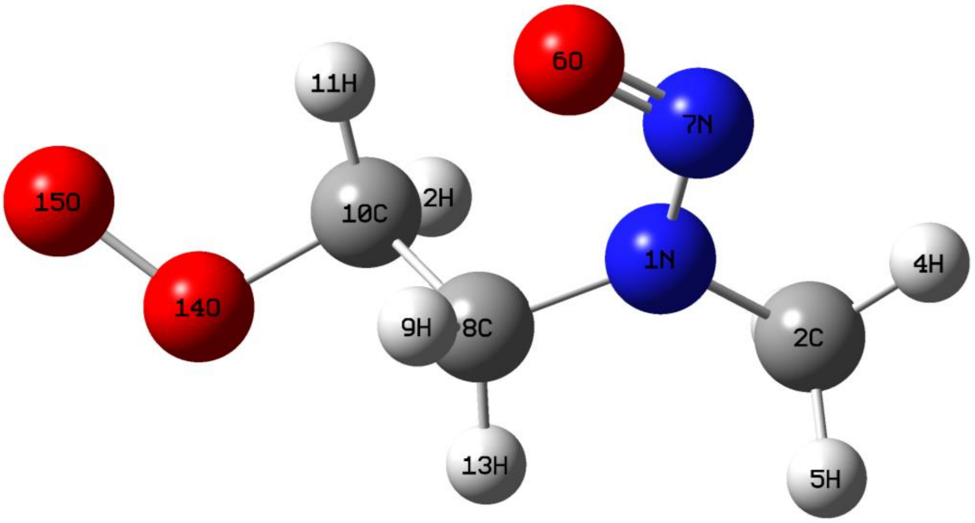

Row	Highlight	Display	Tag	Symbol	NA	NB	NC	Bond	Angle	Dihedral
1	No	Show	1	N						
2	No	Show	2	C	1			1.4485433		
3	No	Show	3	H	2	1		1.0958596	111.2226087	
4	No	Show	4	H	2	1	3	1.0908621	107.3450035	119.3820956
5	No	Show	5	H	2	1	4	1.0958514	111.0185778	119.5540348
6	No	Show	6	O	1	2	4	2.1578147	147.5529145	-0.6884636
7	No	Show	7	N	6	1	2	1.2201443	34.3246045	-0.8915772
8	No	Show	8	C	1	7	6	1.4560585	121.0502111	-2.6925460
9	No	Show	9	H	8	1	7	1.0930302	108.0761032	-38.5029839
10	No	Show	10	C	8	1	7	1.5308759	111.7388457	81.7523387
11	No	Show	11	H	10	8	1	1.0888184	112.0444544	-70.5035867
12	No	Show	12	H	10	8	1	1.0954829	111.4428384	53.7530160
13	No	Show	13	H	8	1	7	1.0928014	108.1724623	-156.9199235

14	No	Show	14	O	10	8	1	1.4529866	107.3743870	170.9059452
15	No	Show	15	O	14	10	8	1.3144352	112.3778313	133.6034651

TS22

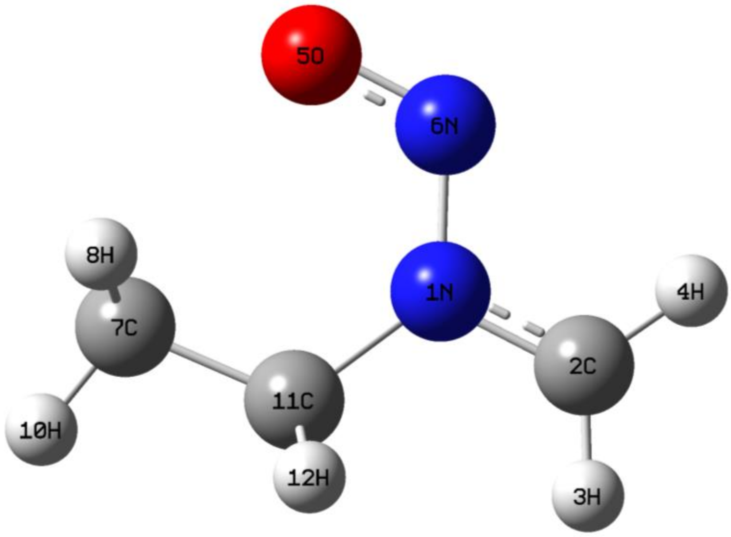

Row	Highlight	Display	Tag	Symbol	NA	NB	NC	Bond	Angle	Dihedral
1	No	Show	1	N						
2	No	Show	2	C	1			1.3335576		
3	No	Show	3	H	2	1		1.0787447	118.9218232	
4	No	Show	4	H	2	1	3	1.0803686	118.1273217	180.0000000
5	No	Show	5	O	1	2	3	2.2234578	145.8482388	180.0000000
6	No	Show	6	N	5	1	2	1.2484082	33.8175881	0.0000000
7	No	Show	7	C	1	2	6	2.6220795	146.4092694	180.0000000
8	No	Show	8	H	7	1	2	1.0897025	96.8966620	124.9248795
9	No	Show	9	H	7	1	2	1.0897025	96.8966620	-124.9248795
10	No	Show	10	H	7	1	2	1.0961768	135.6895637	0.0000000
11	No	Show	11	C	1	2	6	1.5059037	116.2221739	180.0000000
12	No	Show	12	H	11	1	2	1.0924162	104.7086855	56.4345376
13	No	Show	13	H	11	1	2	1.0924154	104.7086966	-56.4345076

TS23

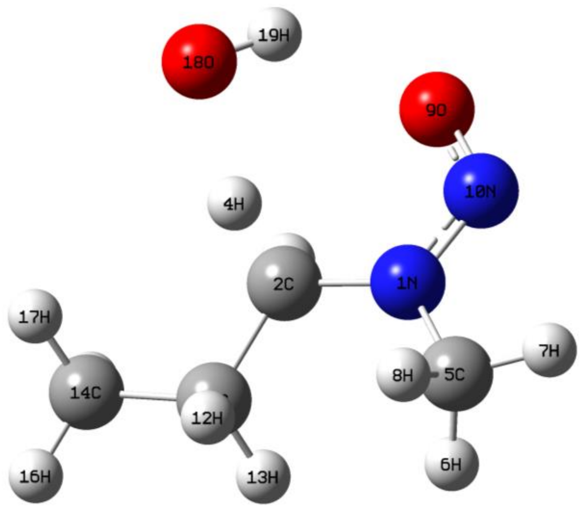

Row	Highlight	Display	Tag	Symbol	NA	NB	NC	Bond	Angle	Dihedral
1	No	Show	1	N						
2	No	Show	2	C	1			1.4509352		
3	No	Show	3	H	2	1		1.0941413	107.8921181	
4	No	Show	4	H	2	1	3	1.1403876	107.3624906	-111.4931526
5	No	Show	5	C	1	2	3	1.4574258	123.4802354	-137.7828341
6	No	Show	6	H	5	1	2	1.0914655	110.4563746	60.5080979
7	No	Show	7	H	5	1	2	1.0894016	107.4361623	-179.8486704
8	No	Show	8	H	5	1	2	1.0929733	110.5247570	-60.5316166
9	No	Show	9	O	1	2	5	2.1612383	89.2385319	-174.9954087
10	No	Show	10	N	9	1	2	1.2435754	33.4008202	179.3909525
11	No	Show	11	C	2	1	10	1.5206172	115.1898822	173.9219741
12	No	Show	12	H	11	2	1	1.0945999	110.2545831	59.2164772
13	No	Show	13	H	11	2	1	1.0968659	109.0170954	-57.9908837
14	No	Show	14	C	11	2	1	1.5294392	110.7479024	-178.8480891
15	No	Show	15	H	14	11	2	1.0951147	111.1712662	60.4290926
16	No	Show	16	H	14	11	2	1.0936403	110.5850808	-179.6845132
17	No	Show	17	H	14	11	2	1.0952179	111.2541026	-59.7341175
18	No	Show	18	O	2	1	10	2.7013810	103.1199465	-49.8972517
19	No	Show	19	H	18	2	1	0.9770852	82.5940115	50.4462648

TS24

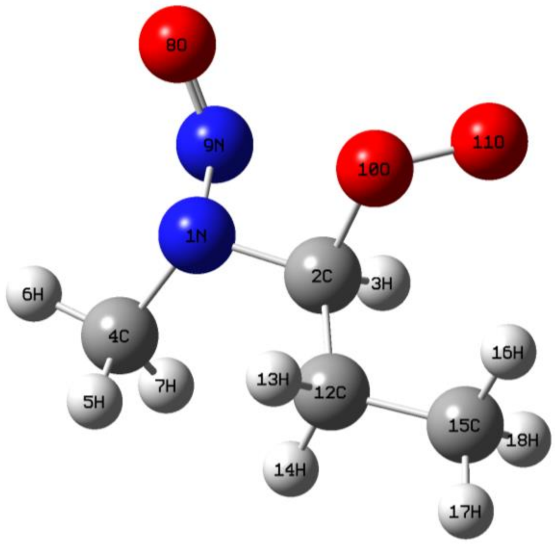

Row	Highlight	Display	Tag	Symbol	NA	NB	NC	Bond	Angle	Dihedral
1	No	Show	1	N						
2	No	Show	2	C	1			1.4502511		
3	No	Show	3	H	2	1		1.0974551	113.1731982	
4	No	Show	4	C	1	2	3	1.4774935	113.0622112	76.1095327
5	No	Show	5	H	4	1	2	1.0900900	108.8329649	73.9972011
6	No	Show	6	H	4	1	2	1.0919959	108.1773559	-169.1577853
7	No	Show	7	H	4	1	2	1.0972283	113.3999897	-48.1880050
8	No	Show	8	O	1	2	4	2.2465458	116.1787950	-139.0562803
9	No	Show	9	N	8	1	2	1.1773496	39.2866375	69.6225795
10	No	Show	10	O	2	1	4	1.4848912	103.3735911	-171.1414053
11	No	Show	11	O	10	2	1	1.3113644	112.5350078	-137.4145101
12	No	Show	12	C	2	1	4	1.5234242	114.6679491	-53.2726554
13	No	Show	13	H	12	2	1	1.0947227	109.0171265	-52.2971483
14	No	Show	14	H	12	2	1	1.0935030	107.4815011	63.8516969
15	No	Show	15	C	12	2	1	1.5301198	112.8433399	-175.6492278
16	No	Show	16	H	15	12	2	1.0933410	111.9393333	62.8911373
17	No	Show	17	H	15	12	2	1.0936335	109.6460180	-177.5662329
18	No	Show	18	H	15	12	2	1.0946475	111.2926797	-58.2124975

TS25

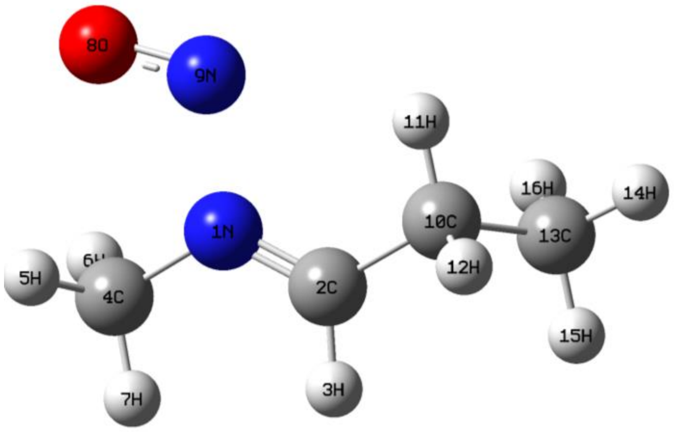

Row	Highlight	Display	Tag	Symbol	NA	NB	NC	Bond	Angle	Dihedral
1	No	Show	1	N						
2	No	Show	2	C	1			1.2800530		
3	No	Show	3	H	2	1		1.0909837	117.8252039	
4	No	Show	4	C	1	2	3	1.4492716	123.5067722	0.0506011
5	No	Show	5	H	4	1	2	1.0922949	109.3138295	125.2809030
6	No	Show	6	H	4	1	2	1.0928402	109.4495158	-116.6296940
7	No	Show	7	H	4	1	2	1.0910219	110.1968604	4.3582781
8	No	Show	8	O	1	2	4	2.3652143	150.6794302	-178.6265627
9	No	Show	9	N	8	1	2	1.2344319	41.8481554	-0.5344998
10	No	Show	10	C	2	1	4	1.4906357	124.3953288	179.0327582
11	No	Show	11	H	10	2	1	1.0893195	110.6197714	5.0931712
12	No	Show	12	H	10	2	1	1.0975184	107.1786536	122.5901890
13	No	Show	13	C	10	2	1	1.5387699	110.9359636	-118.5516321
14	No	Show	14	H	13	10	2	1.0933313	110.0271045	-175.8637748
15	No	Show	15	H	13	10	2	1.0946291	111.1943665	-56.1027478
16	No	Show	16	H	13	10	2	1.0939370	110.9736274	64.3044810

TS26

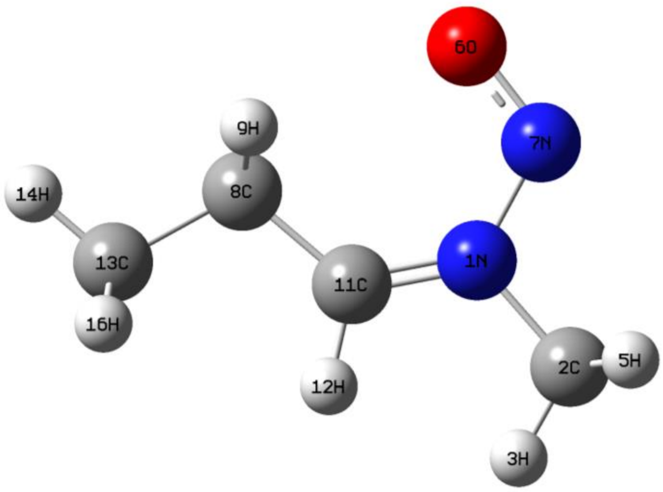

Row	Highlight	Display	Tag	Symbol	NA	NB	NC	Bond	Angle	Dihedral
1	No	Show	1	N						
2	No	Show	2	C	1			1.4700110		
3	No	Show	3	H	2	1		1.0874556	109.4595769	
4	No	Show	4	H	2	1	3	1.0907348	109.1250723	120.2866963
5	No	Show	5	H	2	1	4	1.0907426	109.1346650	119.4151441
6	No	Show	6	O	1	2	3	2.2251143	141.5430828	-179.9948601
7	No	Show	7	N	6	1	2	1.2614998	34.9869942	0.1325482
8	No	Show	8	C	1	7	6	2.5414769	102.0578955	0.1007728
9	No	Show	9	H	8	1	7	1.0952400	96.3824973	-53.3673908
10	No	Show	10	H	8	1	7	1.0950774	96.1842967	52.8709850
11	No	Show	11	C	1	7	6	1.3229992	129.4044870	0.0746888
12	No	Show	12	H	11	1	7	1.0844325	113.5524122	-179.9593411
13	No	Show	13	C	8	1	11	1.5317433	134.5861298	-0.4146474
14	No	Show	14	H	13	8	1	1.0933325	110.3592854	-179.8222906
15	No	Show	15	H	13	8	1	1.0949635	111.1442140	-59.9679033
16	No	Show	16	H	13	8	1	1.0949703	111.1524049	60.3243112

TS27

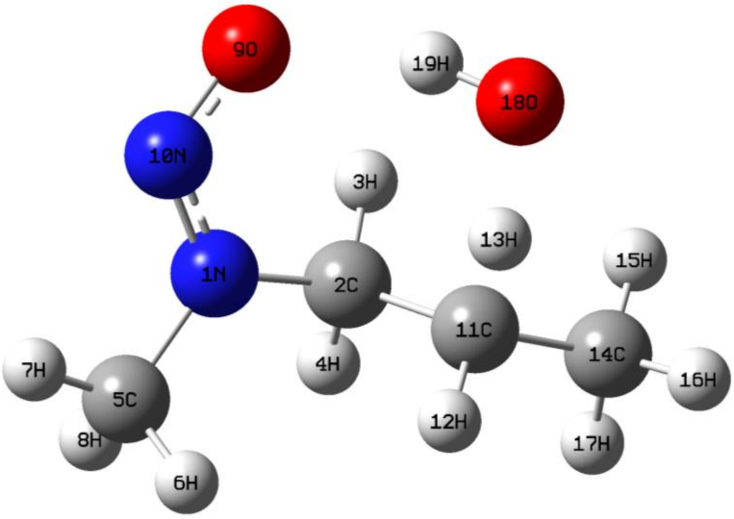

Row	Highlight	Display	Tag	Symbol	NA	NB	NC	Bond	Angle	Dihedral
1	No	Show	1	N						
2	No	Show	2	C	1			1.4661641		
3	No	Show	3	H	2	1		1.0913764	107.4538649	
4	No	Show	4	H	2	1	3	1.0946952	106.3777197	-116.1646102
5	No	Show	5	C	1	2	3	1.4557051	119.7724988	150.0077038
6	No	Show	6	H	5	1	2	1.0932894	109.8401726	58.9229419
7	No	Show	7	H	5	1	2	1.0895143	108.4238943	178.7701658
8	No	Show	8	H	5	1	2	1.0934729	109.9301612	-60.9277052
9	No	Show	9	O	1	5	2	2.1681878	147.7531158	-179.4426658

10	No	Show	10	N	9	1	5	1.2476787	32.6561530	-2.1413312
11	No	Show	11	C	2	1	10	1.5253647	113.7707344	93.8624623
12	No	Show	12	H	11	2	1	1.0953632	111.2393250	47.3145497
13	No	Show	13	H	11	2	1	1.1633792	108.3716780	-66.3415017
14	No	Show	14	C	11	2	1	1.5185013	112.9606706	174.4232466
15	No	Show	15	H	14	11	2	1.0947792	111.4395166	55.7225075
16	No	Show	16	H	14	11	2	1.0934979	110.9270191	176.4803070
17	No	Show	17	H	14	11	2	1.0969493	110.4141230	-64.0720432
18	No	Show	18	O	11	2	1	2.6240399	102.0473566	-70.9266422
19	No	Show	19	H	18	11	2	0.9800623	87.5536003	7.4694241

TS28

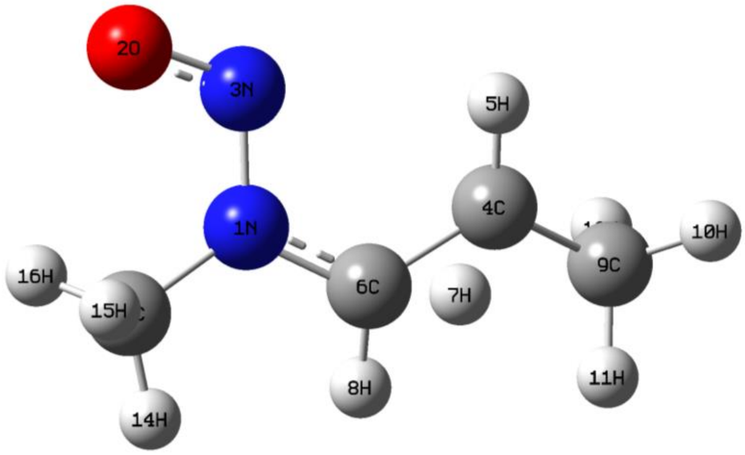

Row	Highlight	Display	Tag	Symbol	NA	NB	NC	Bond	Angle	Dihedral
1	No	Show	1	N						
2	No	Show	2	O	1			2.1902917		
3	No	Show	3	N	2	1		1.2701494	34.7746493	
4	No	Show	4	C	1	3	2	2.4920924	88.9806580	-172.7551769
5	No	Show	5	H	4	1	3	1.0804054	90.9513035	13.3715154
6	No	Show	6	C	1	3	2	1.3772106	118.2368988	-172.7977567
7	No	Show	7	H	6	1	3	1.2432577	116.1098062	73.4550998
8	No	Show	8	H	6	1	3	1.0841389	115.3890841	-160.1810070
9	No	Show	9	C	4	1	3	1.4925212	146.0605776	-148.9741297
10	No	Show	10	H	9	4	1	1.0933738	111.7308124	-161.9276387
11	No	Show	11	H	9	4	1	1.0949058	111.6549700	-39.9879940
12	No	Show	12	H	9	4	1	1.1003372	110.2095896	78.7927231
13	No	Show	13	C	1	3	2	1.4598598	120.6535582	-0.1544492
14	No	Show	14	H	13	1	3	1.0889866	108.4080032	175.7068896
15	No	Show	15	H	13	1	3	1.0936251	110.7511587	-63.9184404
16	No	Show	16	H	13	1	3	1.0914043	109.5510804	55.6549231

TS29

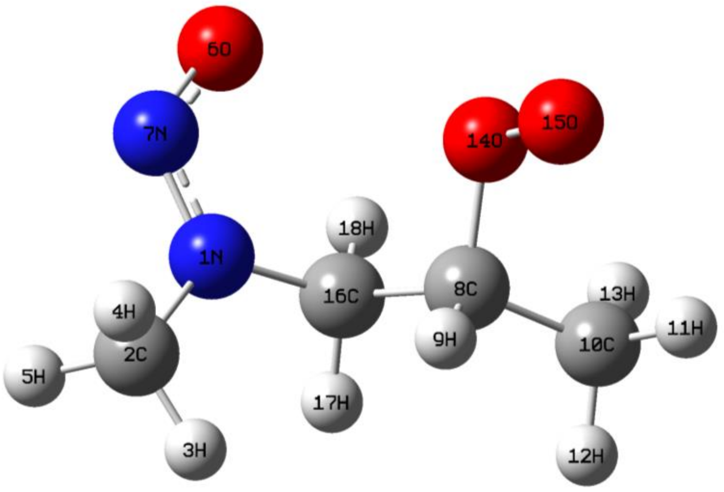

Row	Highlight	Display	Tag	Symbol	NA	NB	NC	Bond	Angle	Dihedral
1	No	Show	1	N						
2	No	Show	2	C	1			1.4637164		
3	No	Show	3	H	2	1		1.0891124	108.4828733	
4	No	Show	4	H	2	1	3	1.0917625	110.5244416	119.4089977
5	No	Show	5	H	2	1	3	1.0925388	110.5344423	-119.3225875
6	No	Show	6	O	1	2	3	2.1698517	146.3259634	-175.5419264
7	No	Show	7	N	6	1	2	1.2384740	32.8005625	-3.2705666
8	No	Show	8	C	1	7	6	2.4997612	121.8164641	39.7564653
9	No	Show	9	H	8	1	7	1.0921572	88.3309034	120.1540248
10	No	Show	10	C	8	1	7	1.5177436	144.7992102	-113.2124426
11	No	Show	11	H	10	8	1	1.0924715	109.9511882	-179.6435676
12	No	Show	12	H	10	8	1	1.0933729	109.4327242	-60.5418330
13	No	Show	13	H	10	8	1	1.0939796	111.0313983	59.4450778
14	No	Show	14	O	8	1	7	1.4784493	91.6342371	14.6399627
15	No	Show	15	O	14	8	1	1.3102603	111.9432045	131.4824272
16	No	Show	16	C	1	7	6	1.4624390	123.0167562	-0.7126657
17	No	Show	17	H	16	1	7	1.0916277	107.8177370	-140.8570788
18	No	Show	18	H	16	1	7	1.0903554	107.7692860	-23.3413597

TS31

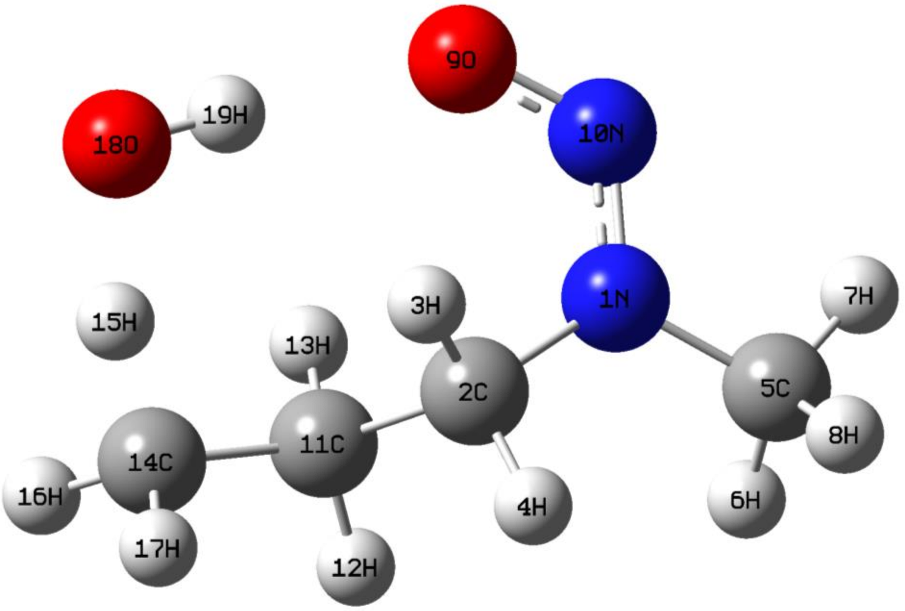

Row	Highlight	Display	Tag	Symbol	NA	NB	NC	Bond	Angle	Dihedral
1	No	Show	1	N						
2	No	Show	2	C	1			1.4634401		
3	No	Show	3	H	2	1		1.0922191	107.0464696	
4	No	Show	4	H	2	1	3	1.0905810	107.0527621	-115.7426915
5	No	Show	5	C	1	2	4	1.4553100	121.0582296	21.6629014
6	No	Show	6	H	5	1	2	1.0933771	109.9141629	57.4989885
7	No	Show	7	H	5	1	2	1.0898414	108.3534529	177.4469928
8	No	Show	8	H	5	1	2	1.0935985	110.0742649	-62.4838881
9	No	Show	9	O	1	5	2	2.1652198	148.3162049	-178.5735390
10	No	Show	10	N	9	1	5	1.2475625	32.7915550	-1.5483163
11	No	Show	11	C	2	1	10	1.5408239	112.8007185	78.3225114
12	No	Show	12	H	11	2	1	1.0968594	109.5700688	83.7857989
13	No	Show	13	H	11	2	1	1.0934649	109.7051843	-33.0975018
14	No	Show	14	C	11	2	1	1.5146714	111.6467431	-154.1129743
15	No	Show	15	H	14	11	2	1.1931570	107.7497443	63.2779574
16	No	Show	16	H	14	11	2	1.0925576	113.5912893	178.7343458
17	No	Show	17	H	14	11	2	1.0939294	114.3073940	-52.3676278
18	No	Show	18	O	14	11	2	2.5713879	99.2220398	62.8013999
19	No	Show	19	H	18	14	11	0.9789974	88.9850247	1.1260975

TS32

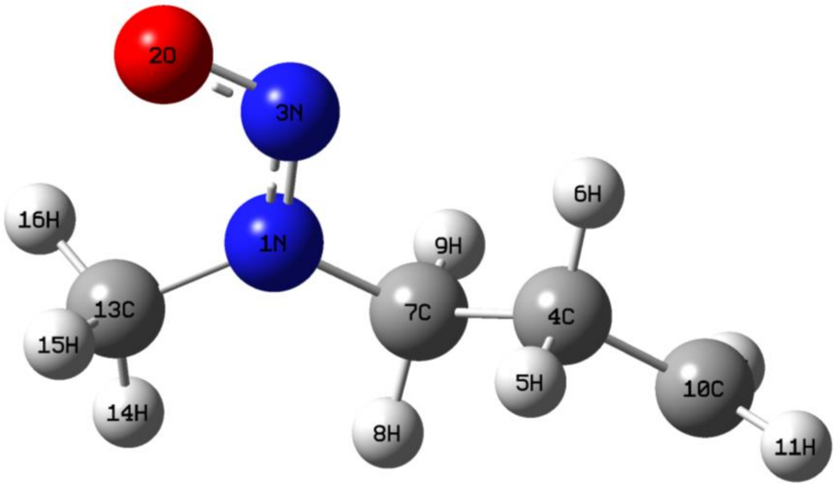

Row	Highlight	Display	Tag	Symbol	NA	NB	NC	Bond	Angle	Dihedral
1	No	Show	1	N						
2	No	Show	2	O	1			2.1526737		
3	No	Show	3	N	2	1		1.2451945	33.2925211	
4	No	Show	4	C	1	3	2	2.5165380	91.6622689	-159.6102510
5	No	Show	5	H	4	1	3	1.0964851	90.3446368	90.3812761
6	No	Show	6	H	4	1	3	1.1012968	90.3474875	-15.4400192
7	No	Show	7	C	1	3	2	1.4716070	117.4387039	177.3775125
8	No	Show	8	H	7	1	3	1.0902995	106.2943757	165.2885170
9	No	Show	9	H	7	1	3	1.0959937	109.1868290	-78.9974301
10	No	Show	10	C	4	1	3	1.4877426	143.7503204	-142.5283250
11	No	Show	11	H	10	4	1	1.0837579	120.5164672	-153.0416723
12	No	Show	12	H	10	4	1	1.0853649	120.6739768	36.8804216
13	No	Show	13	C	1	3	2	1.4601204	120.4367340	2.1339873
14	No	Show	14	H	13	1	3	1.0876642	108.5118531	176.1905382
15	No	Show	15	H	13	1	3	1.0937285	110.1531551	-63.4019459
16	No	Show	16	H	13	1	3	1.0935557	109.9657754	55.7940244

TS33

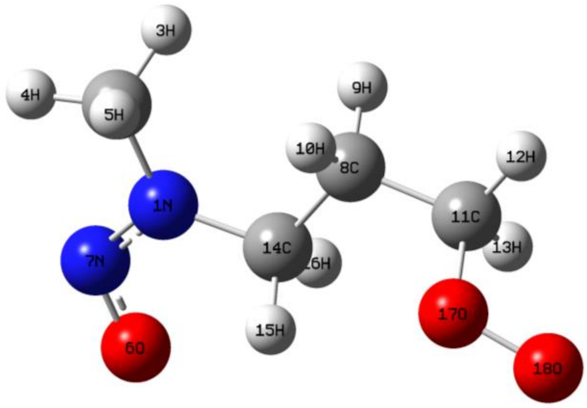

Row	Highlight	Display	Tag	Symbol	NA	NB	NC	Bond	Angle	Dihedral
1	No	Show	1	N						

2	No	Show	2	C	1			1.4561016		
3	No	Show	3	H	2	1		1.0922147	110.8154809	
4	No	Show	4	H	2	1	3	1.0896050	107.3500979	119.4026044
5	No	Show	5	H	2	1	4	1.0930188	110.7118426	119.2579560
6	No	Show	6	O	1	2	4	2.1558576	147.5786647	-3.7363575
7	No	Show	7	N	6	1	2	1.2432460	33.3700593	2.8956772
8	No	Show	8	C	1	7	6	2.5066427	153.4650141	7.4698026
9	No	Show	9	H	8	1	7	1.0942404	93.7061345	111.6909824
10	No	Show	10	H	8	1	7	1.0940745	91.9998031	-140.7061056
11	No	Show	11	C	8	1	7	1.5169171	143.8919264	-14.2090127
12	No	Show	12	H	11	8	1	1.0920617	111.7457184	176.1402662
13	No	Show	13	H	11	8	1	1.0925208	113.2399474	51.3264983
14	No	Show	14	C	1	7	6	1.4691963	119.9112003	-0.2380131
15	No	Show	15	H	14	1	7	1.0955613	107.9045322	-62.8715012
16	No	Show	16	H	14	1	7	1.0948964	107.3716016	51.3537663
17	No	Show	17	O	11	8	1	1.4674058	107.7423747	-66.8263071
18	No	Show	18	O	17	11	8	1.3101564	112.0202137	173.7779652

TS34=TS28

TS35

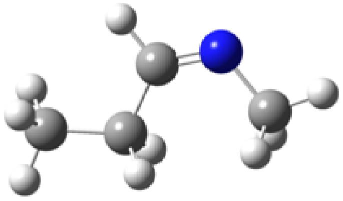

TS36

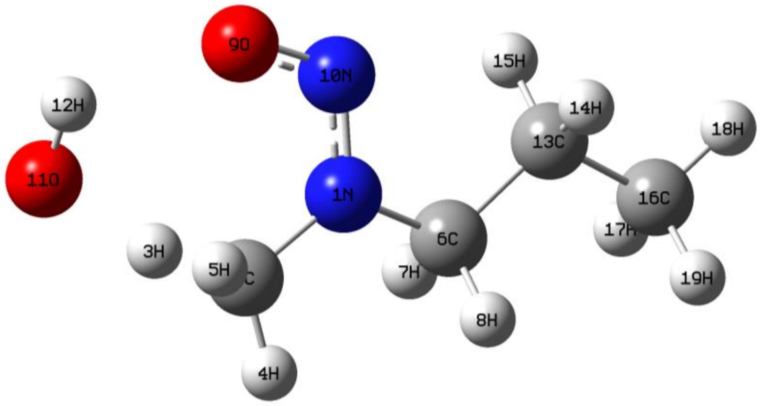

Row	Highlight	Display	Tag	Symbol	NA	NB	NC	Bond	Angle	Dihedral
1	No	Show	1	N						
2	No	Show	2	C	1			1.4303963		
3	No	Show	3	H	2	1		1.1691815	109.4255091	
4	No	Show	4	H	2	1	3	1.0882755	110.2618641	118.0764983

5	No	Show	5	H	2	1	4	1.0908187	111.5701252	126.3301748
6	No	Show	6	C	1	2	4	1.4717076	120.1598414	8.7451413
7	No	Show	7	H	6	1	2	1.0962890	107.1390408	59.7087927
8	No	Show	8	H	6	1	2	1.0945403	106.7109809	-54.8404867
9	No	Show	9	O	1	2	6	2.1545654	89.1363904	174.3937063
10	No	Show	10	N	9	1	2	1.2435462	33.6853335	-179.7798586
11	No	Show	11	O	2	1	10	2.5993487	105.1548711	53.8999032
12	No	Show	12	H	11	2	1	0.9755007	85.2008735	-53.9479400
13	No	Show	13	C	6	1	10	1.5237691	114.6515527	8.2765520
14	No	Show	14	H	13	6	1	1.0947542	109.7155511	57.6338222
15	No	Show	15	H	13	6	1	1.0938715	109.9577441	-59.6645686
16	No	Show	16	C	13	6	1	1.5305744	110.2711610	178.9266653
17	No	Show	17	H	16	13	6	1.0953077	111.3005488	60.8518070
18	No	Show	18	H	16	13	6	1.0938048	110.4944155	-179.3567397
19	No	Show	19	H	16	13	6	1.0954045	111.3845681	-59.5279643

TS37

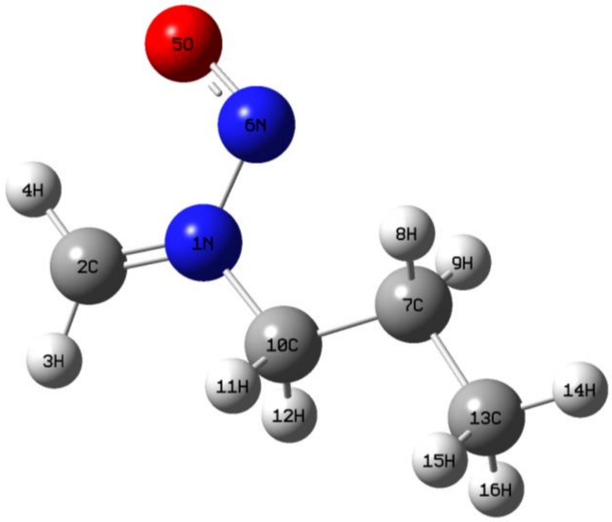

Row	Highlight	Display	Tag	Symbol	NA	NB	NC	Bond	Angle	Dihedral
1	No	Show	1	N						
2	No	Show	2	C	1			1.3198112		
3	No	Show	3	H	2	1		1.0798664	118.6665614	
4	No	Show	4	H	2	1	3	1.0789241	118.2734126	180.0000000
5	No	Show	5	O	1	2	4	2.1976701	91.4856279	0.0000000
6	No	Show	6	N	5	1	2	1.2518474	35.6769024	-180.0000000
7	No	Show	7	C	1	2	6	2.5360298	153.4919253	-179.9991566
8	No	Show	8	H	7	1	2	1.0935999	91.7867037	-126.4632383
9	No	Show	9	H	7	1	2	1.0936007	91.7864141	126.4616117
10	No	Show	10	C	1	2	6	1.4827772	120.4856668	-180.0000000
11	No	Show	11	H	10	1	2	1.0934511	106.6983427	-57.4107801

12	No	Show	12	H	10	1	2	1.0934517	106.6982742	57.4098410
13	No	Show	13	C	7	1	2	1.5310397	141.9281887	-0.0005498
14	No	Show	14	H	13	7	1	1.0939858	110.4900677	179.9995246
15	No	Show	15	H	13	7	1	1.0955134	111.4115100	-60.2090559
16	No	Show	16	H	13	7	1	1.0955136	111.4115102	60.2080481

TS38

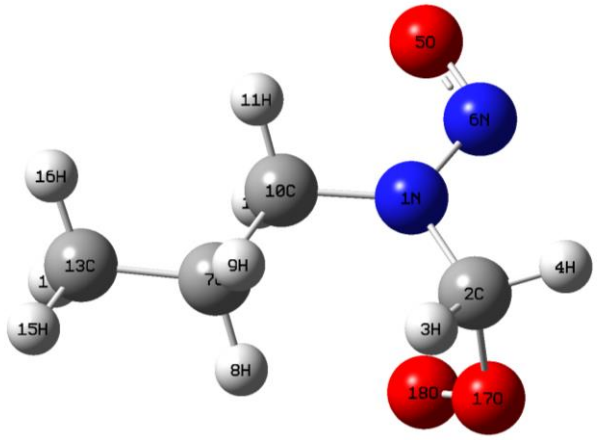

Row	Highlight	Display	Tag	Symbol	NA	NB	NC	Bond	Angle	Dihedral
1	No	Show	1	N						
2	No	Show	2	C	1			1.4126208		
3	No	Show	3	H	2	1		1.0875081	112.5762888	
4	No	Show	4	H	2	1	3	1.0887112	110.2356991	-125.1668968
5	No	Show	5	O	1	2	3	2.1588920	144.6581258	155.9439326
6	No	Show	6	N	5	1	2	1.2251456	34.2125017	-9.4887632
7	No	Show	7	C	1	6	5	2.5142356	153.6038233	-3.7554274
8	No	Show	8	H	7	1	6	1.0951232	91.7075127	136.7266763
9	No	Show	9	H	7	1	6	1.0955970	91.6251158	-115.8876477
10	No	Show	10	C	1	6	5	1.4740898	120.2162645	2.7517279
11	No	Show	11	H	10	1	6	1.0941976	106.6118741	-52.6487932
12	No	Show	12	H	10	1	6	1.0952712	107.5671192	61.7392870
13	No	Show	13	C	7	1	6	1.5302757	142.8754428	10.4962768
14	No	Show	14	H	13	7	1	1.0951731	111.2422675	60.0655715
15	No	Show	15	H	13	7	1	1.0938013	110.5270969	179.9345450
16	No	Show	16	H	13	7	1	1.0953519	111.3154832	-60.2489474
17	No	Show	17	O	2	1	6	1.5020762	113.6128665	-90.6567910
18	No	Show	18	O	17	2	1	1.3047221	114.2828433	-10.6530635

TS39

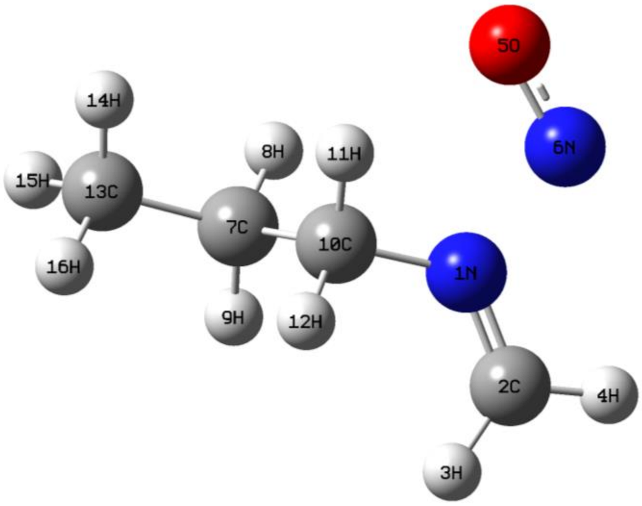

Row	Highlight	Display	Tag	Symbol	NA	NB	NC	Bond	Angle	Dihedral
1	No	Show	1	N						
2	No	Show	2	C	1			1.2725018		
3	No	Show	3	H	2	1		1.0866470	121.5948551	
4	No	Show	4	H	2	1	3	1.0876169	118.1789552	-179.7733985
5	No	Show	5	O	1	2	3	2.3875984	146.2422880	179.5003000
6	No	Show	6	N	5	1	2	1.2274691	41.5661996	1.4934893
7	No	Show	7	C	1	2	6	2.4692123	117.6961869	137.4357158
8	No	Show	8	H	7	1	2	1.0951423	88.9623956	-119.4032784
9	No	Show	9	H	7	1	2	1.0960589	90.6699810	-12.2972426
10	No	Show	10	C	1	2	6	1.4529609	125.0388141	178.1186323
11	No	Show	11	H	10	1	2	1.0948155	106.2111942	150.6967086
12	No	Show	12	H	10	1	2	1.0934057	108.7609868	33.6470900
13	No	Show	13	C	7	1	2	1.5283500	144.6494318	115.7411966
14	No	Show	14	H	13	7	1	1.0953141	111.2891700	58.6060740
15	No	Show	15	H	13	7	1	1.0940796	110.6851876	178.5030168
16	No	Show	16	H	13	7	1	1.0955517	111.2859758	-61.6352456

TS40

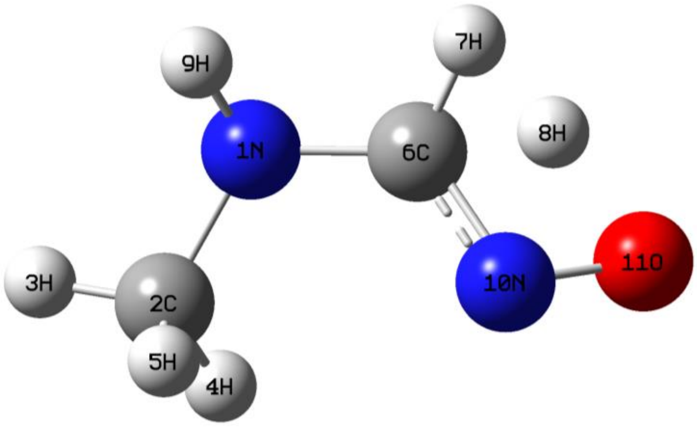

Row	Highlight	Display	Tag	Symbol	NA	NB	NC	Bond	Angle	Dihedral

1	No	Show	1	N						
2	No	Show	2	C	1			1.4587018		
3	No	Show	3	H	2	1		1.0921032	108.8830442	
4	No	Show	4	H	2	1	3	1.0940768	109.1781127	118.4610692
5	No	Show	5	H	2	1	3	1.0972885	113.6892615	-120.9062169
6	No	Show	6	C	1	2	3	1.3873169	118.4620025	-169.3147049
7	No	Show	7	H	6	1	2	1.0949470	114.7973465	-154.5795697
8	No	Show	8	H	6	1	2	1.3929482	138.5292966	105.2441159
9	No	Show	9	H	1	6	2	1.0133792	113.6286602	136.2252305
10	No	Show	10	N	6	1	2	1.3602468	124.9841055	-11.6908964
11	No	Show	11	O	10	6	1	1.3139965	103.4771574	149.3563145

TS42

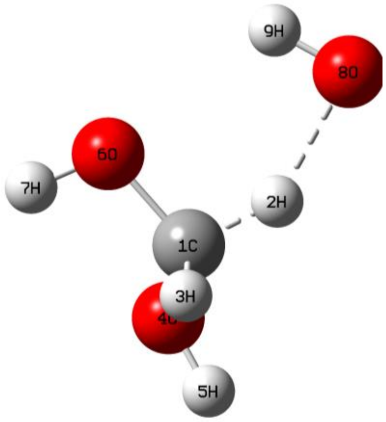

Row	Highlight	Display	Tag	Symbol	NA	NB	NC	Bond	Angle	Dihedral
1	No	Show	1	C						
2	No	Show	2	H	1			1.1260983		
3	No	Show	3	H	1	2		1.1058467	107.4897255	
4	No	Show	4	O	1	3	2	1.3999987	111.0770412	122.6459082
5	No	Show	5	H	4	1	3	0.9632630	108.8326159	-40.2364282
6	No	Show	6	O	1	4	5	1.3890382	108.9016010	-164.6490202
7	No	Show	7	H	6	1	4	0.9645693	107.7409985	49.8656962
8	No	Show	8	O	1	6	4	2.6920396	84.0972988	124.0469633
9	No	Show	9	H	8	1	6	0.9744265	76.3661559	-5.0361799

TS43

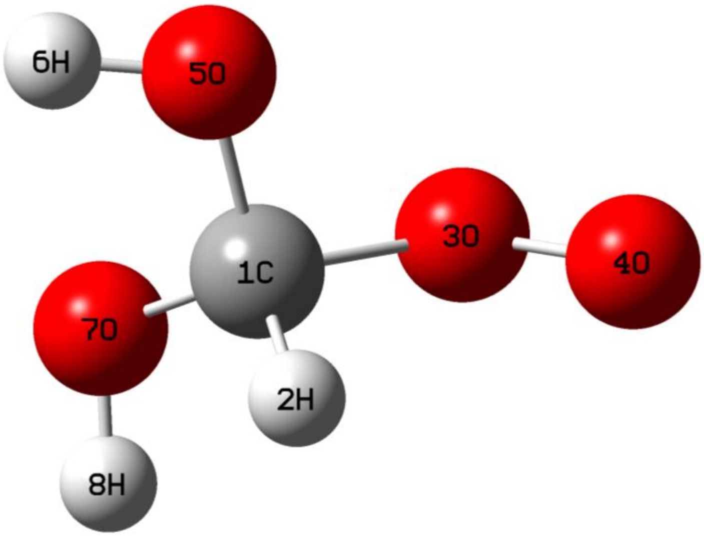

Row	Highlight	Display	Tag	Symbol	NA	NB	NC	Bond	Angle	Dihedral
1	No	Show	1	C						
2	No	Show	2	H	1			1.0950145		
3	No	Show	3	O	1	2		1.4891424	104.9478713	
4	No	Show	4	O	3	1	2	1.3119434	111.0267162	-33.6337657
5	No	Show	5	O	1	3	4	1.3796067	104.8922413	86.8687018
6	No	Show	6	H	5	1	3	0.9673515	107.7510176	148.5778547
7	No	Show	7	O	1	5	3	1.3691767	110.4291964	-115.1093192
8	No	Show	8	H	7	1	5	0.9674666	108.9908847	-160.7617545

TS44

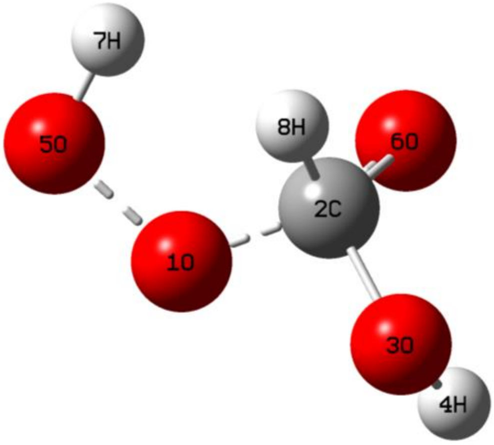

Row	Highlight	Display	Tag	Symbol	NA	NB	NC	Bond	Angle	Dihedral
1	No	Show	1	O						
2	No	Show	2	C	1			1.4778664		
3	No	Show	3	O	2	1		1.3733333	107.7530404	
4	No	Show	4	H	3	2	1	0.9703008	107.6711256	74.6589445
5	No	Show	5	O	1	2	3	1.4192941	113.3318805	148.2526008
6	No	Show	6	O	2	1	5	1.3440994	96.6870029	-91.4846567

7	No	Show	7	H	5	1	2	0.9747164	105.1947390	20.6648108
8	No	Show	8	H	2	1	5	1.0968676	108.8880392	30.5923668

TS45

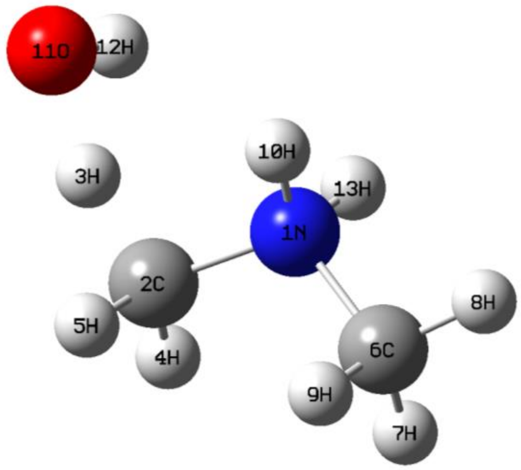

Row	Highlight	Display	Tag	Symbol	NA	NB	NC	Bond	Angle	Dihedral
1	No	Show	1	N						
2	No	Show	2	C	1			1.4765832		
3	No	Show	3	H	2	1		1.2310653	106.8814092	
4	No	Show	4	H	2	1	3	1.0876667	111.0080794	117.3646271
5	No	Show	5	H	2	1	4	1.0890055	110.7158004	128.6950154
6	No	Show	6	C	1	2	4	1.5003489	113.3113234	-67.5202902
7	No	Show	7	H	6	1	2	1.0891874	108.3365711	61.0319131
8	No	Show	8	H	6	1	2	1.0894204	108.0745001	-179.0289128
9	No	Show	9	H	6	1	2	1.0891052	108.1664610	-59.1288467
10	No	Show	10	H	1	2	6	1.0241826	108.9936541	-121.7456967
11	No	Show	11	O	2	1	6	2.4913759	100.0533370	177.6741696
12	No	Show	12	H	11	2	1	0.9736109	94.4453685	73.0574127
13	No	Show	13	H	1	2	6	1.0241006	109.0557445	121.9436637

TS46

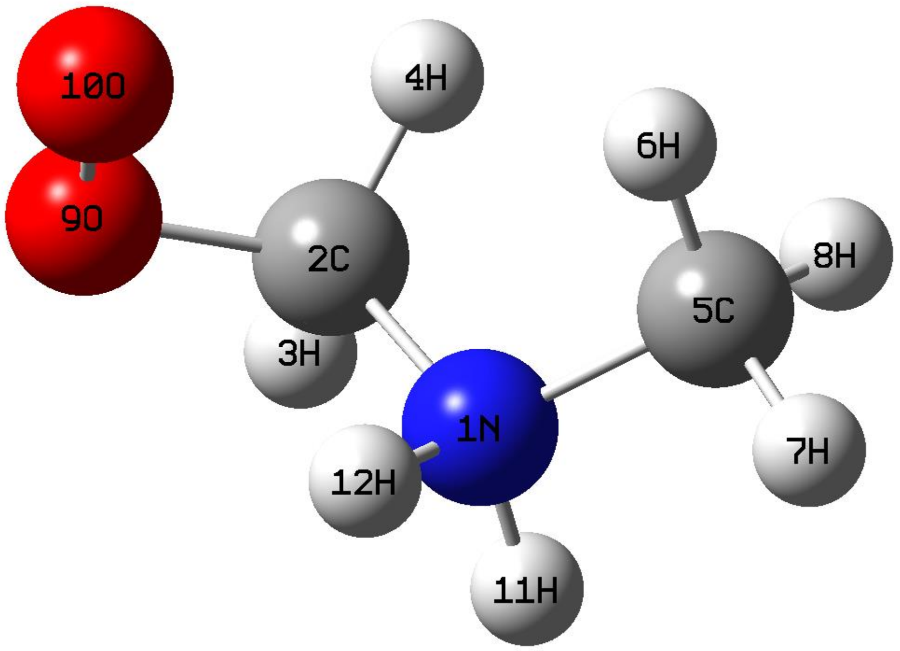

Row	Highlight	Display	Tag	Symbol	NA	NB	NC	Bond	Angle	Dihedral
1	No	Show	1	N						
2	No	Show	2	C	1			1.5016009		
3	No	Show	3	H	2	1		1.0875203	109.5701672	
4	No	Show	4	H	2	1	3	1.0879885	109.6774652	-124.0622994
5	No	Show	5	C	1	2	3	1.4999029	114.4170157	125.6096364
6	No	Show	6	H	5	1	2	1.0886930	107.9789970	61.1720061
7	No	Show	7	H	5	1	2	1.0888202	107.8464523	-178.8636944
8	No	Show	8	H	5	1	2	1.0891212	108.1595353	-59.1377955
9	No	Show	9	O	2	1	5	1.4315889	109.5771930	-118.8517407
10	No	Show	10	O	9	2	1	1.3181016	111.1902110	67.0223512
11	No	Show	11	H	1	5	2	1.0239958	109.2463450	122.2394570
12	No	Show	12	H	1	5	2	1.0258707	108.8909892	-121.2371914

TS47

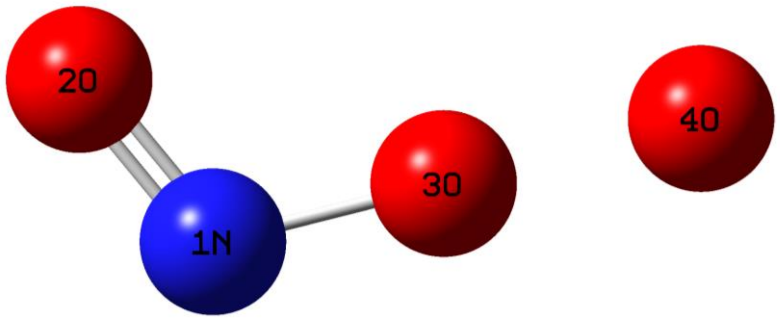

Row	Highlight	Display	Tag	Symbol	NA	NB	NC	Bond	Angle	Dihedral
1	No	Show	1	N						
2	No	Show	2	O	1			1.1888592		

3	No	Show	3	O	1	2		1.3467784	115.4163176		
4	No	Show	4	O	3	1	2	1.5030434	179.9988663	89.9085640	

TS48

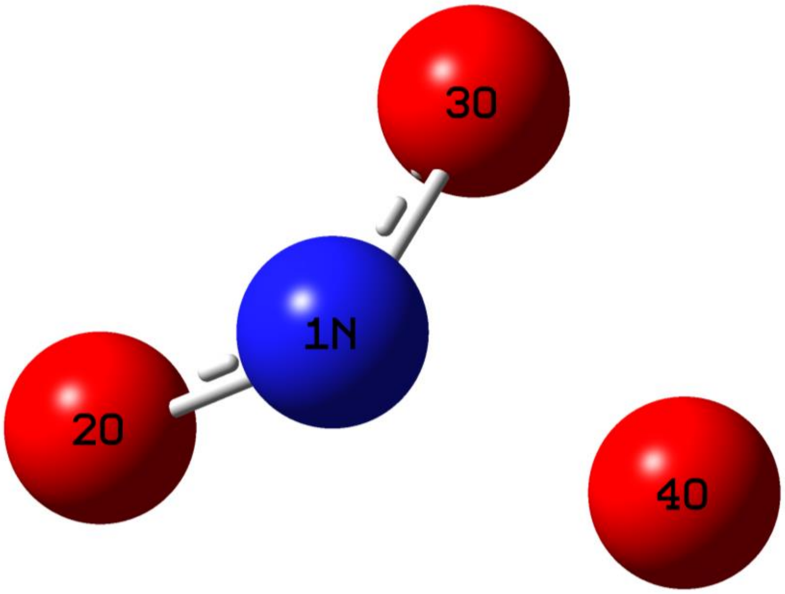

Row	Highlight	Display	Tag	Symbol	NA	NB	NC	Bond	Angle	Dihedral
1	No	Show	1	N						
2	No	Show	2	O	1			1.2280958		
3	No	Show	3	O	1	2		1.2783238	117.1880396	
4	No	Show	4	O	1	2	3	1.7330686	116.9003351	-89.2426082

TS49

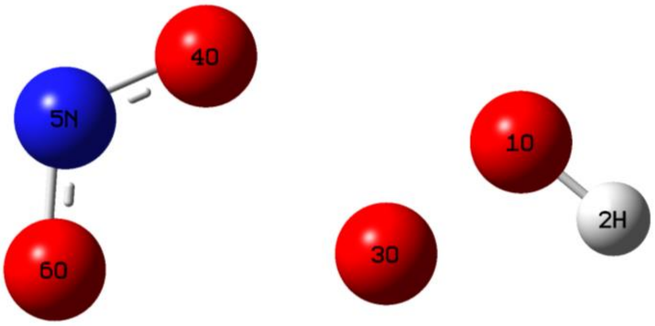

Row	Highlight	Display	Tag	Symbol	NA	NB	NC	Bond	Angle	Dihedral
1	No	Show	1	O						
2	No	Show	2	H	1			0.9741814		
3	No	Show	3	O	1	2		1.4192722	100.8181308	
4	No	Show	4	O	3	1	2	2.1663143	92.9095041	-179.9834816
5	No	Show	5	N	4	3	1	1.2475313	108.7886270	-179.5286391
6	No	Show	6	O	5	4	3	1.2299882	117.5899412	-0.1442372

TS50

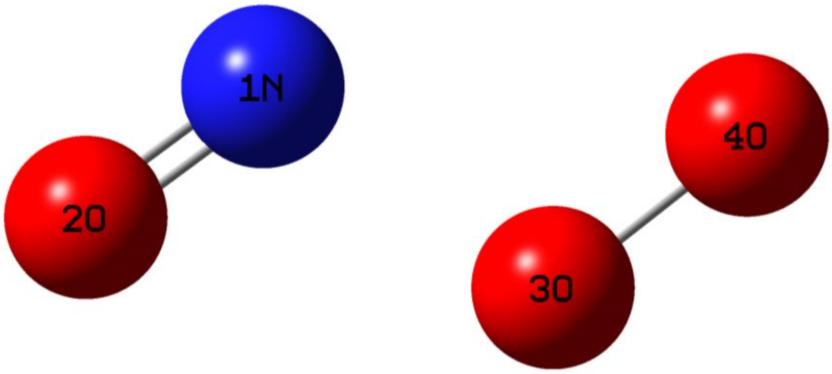

Row	Highlight	Display	Tag	Symbol	NA	NB	NC	Bond	Angle	Dihedral
1	No	Show	1	N						
2	No	Show	2	O	1			1.1181846		
3	No	Show	3	O	1	2		1.7925161	108.8293135	
4	No	Show	4	O	3	1	2	1.2536053	107.1008677	-179.9622208

TS51

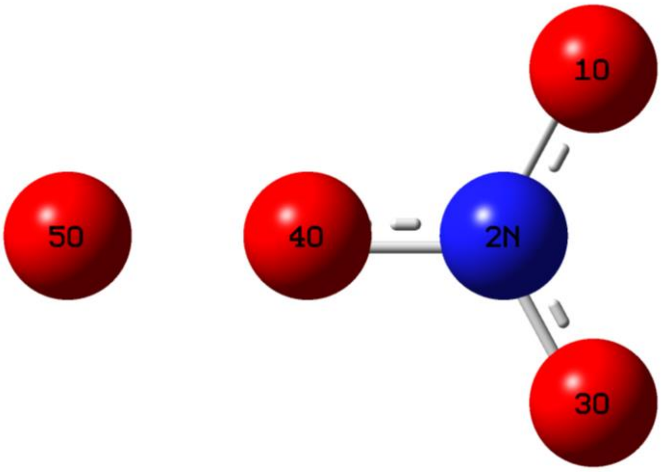

Row	Highlight	Display	Tag	Symbol	NA	NB	NC	Bond	Angle	Dihedral
1	No	Show	1	O						
2	No	Show	2	N	1			1.2256005		
3	No	Show	3	O	2	1		1.2256006	123.4897937	
4	No	Show	4	O	2	1	3	1.2710450	118.2542113	-179.9967222
5	No	Show	5	O	4	2	1	1.5530690	179.9977953	-70.7925088

TS52

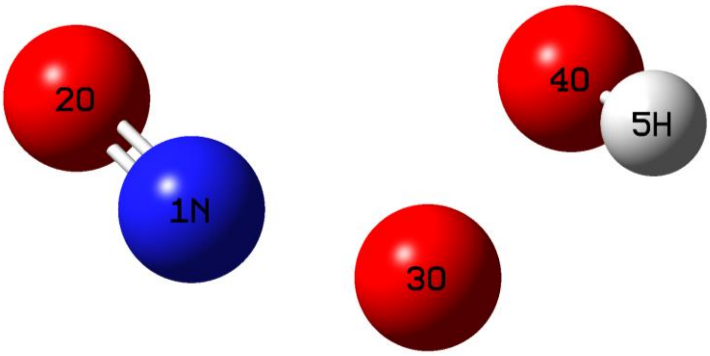

Row	Highlight	Display	Tag	Symbol	NA	NB	NC	Bond	Angle	Dihedral
1	No	Show	1	N						
2	No	Show	2	O	1			1.1395924		
3	No	Show	3	O	1	2		1.5768614	109.1151508	
4	No	Show	4	O	3	1	2	1.4077887	101.9529810	85.6588239
5	No	Show	5	H	4	3	1	0.9757984	102.6976776	91.3593034

TS53

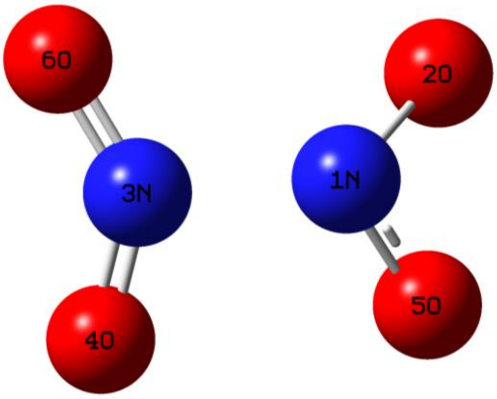

Row	Highlight	Display	Tag	Symbol	NA	NB	NC	Bond	Angle	Dihedral
1	No	Show	1	N						
2	No	Show	2	O	1			1.3889452		
3	No	Show	3	N	1	2		1.6655300	109.1595436	
4	No	Show	4	O	3	1	2	1.1815411	110.2798928	118.4276055
5	No	Show	5	O	1	2	3	1.2391592	87.0850850	108.2438695
6	No	Show	6	O	3	1	5	1.1825345	115.2001144	-158.4639506

© 2018 by the authors. Submitted for possible open access publication under the terms and conditions of the Creative Commons Attribution (CC BY) license (http://creativecommons.org/licenses/by/4.0/).
